# 15-year trends in efficacy and effectiveness of treatment outcomes in drug-resistant pulmonary TB

**DOI:** 10.5588/ijtldopen.24.0620

**Published:** 2025-04-09

**Authors:** M.J. Nasiri, M. Amiri, M. Cheraghi, D.R. Silva, G. Sotgiu, L. D’Ambrosio, R. Centis, M. Mileva-Lopez, T.M. Hill, S. Gidey, K. Diaby, N. Hittel, H. Gandhi, M. Dara

**Affiliations:** ^1^ Department of Microbiology, School of Medicine, Shahid Beheshti University of Medical Sciences, Tehran, Iran;; ^2^ Faculdade de Medicina, Universidade Federal do Rio Grande do Sul (UFRGS), Porto Alegre, Brazil;; ^3^ Clinical Epidemiology and Medical Statistics Unit, Department of Medicine, Surgery and Pharmacy, University of Sassari, Sassari, Italy;; ^4^ Public Health Consulting Group, Lugano, Switzerland;; ^5^ Servizio di Epidemiologia Clinica delle Malattie Respiratorie, Istituti Clinici Scientifici Maugeri, IRCCS, Tradate, Italy;; ^6^ Otsuka Novel Products, Munich, Germany;; ^7^ Otsuka Pharmaceutical Development & Commercialization, Rockville, MD, USA.

**Keywords:** DR-TB, systematic review, meta-analysis, efficacy, effectiveness, outcomes

## Abstract

**BACKGROUND:**

This study describes the evolution of treatment outcomes in drug-resistant (DR) pulmonary TB, focusing on efficacy and effectiveness.

**METHODS:**

We searched PubMed/MEDLINE, Embase, Cochrane CENTRAL, Scopus, and Web of Science reporting DR-TB regimens from 1 January 2009 to 8 May 2024 and performed a systematic literature review and meta-analysis.

**RESULTS:**

A gradual increase in success rates in the treatment of DR pulmonary TB was observed from 2009 to 2024 across all studies. In observational studies, the average treatment success rate for mono-resistant TB (non-rifampicin-resistant TB, RR-TB) was 82.9%, while the average treatment success rate for RR/multidrug-resistant TB (MDR-TB) was 68.4%, and that of pre-extensively drug-resistant TB (pre-XDR-TB) and XDR-TB was 54.4% with an increasing trend over time. The outcomes of experimental studies, which included fewer patients, demonstrated 69.6% treatment success for RR/MDR-TB, with higher rates for pre-XDR/XDR-TB (79.2%) and a mix of the two groups (85.8%). Significant geographic variations in outcome rates were observed across studies.

**CONCLUSION:**

The current study demonstrates a steady improvement in treatment outcomes for DR-TB after a long period of stagnation. However, new drugs and novel regimens are needed to maintain or further improve treatment outcomes in DR-TB.

TB continues to be a clinical and global public health emergency, with 10.8 million incident TB cases and 1.25 million deaths, including 1.09 million among HIV-negative people and 161,000 among people with HIV. In 2023, there were 175,923 cases of rifampicin-resistant TB or multidrug-resistant TB (RR/MDR-TB) diagnosed and treated worldwide.^1^ The clinical and public health management of the disease is further complicated by the emergence of pre-extensively drug-resistant and extensively drug-resistant TB (XDR-TB).^1,2^

In the past decade, there have been some important improvements in treatment options for drug-resistant TB (DR-TB). After a gap of nearly 40 years, two drugs – bedaquiline (BDQ) and delamanid (DLM), both with novel mechanisms of action – were approved for the treatment of RR/MDR-TB, followed by pretomanid (Pa), recommended as a part of treatment regimens^3^ Concurrently, shorter all-oral regimens have been developed to replace the existing longer regimens.^1,3,4^ There are several constraints in managing DR-TB, including difficulties in providing rapid and accurate diagnosis, designing effective regimens, particularly for XDR-TB, as well as managing frequent and severe adverse events, e.g., those associated with linezolid (LZD) among Group A drugs.^5^

Although extensive literature on DR-TB exists, there is a lack of a comprehensive systematic review and meta-analysis examining the evolution of regimens and their outcomes.

The aim of the present systematic literature review (SLR) and meta-analysis is to describe the evolution of efficacy/effectiveness of the regimens to treat RR/MDR-TB.

## METHODS

### Search strategy

We searched PubMed/MEDLINE, Embase, Cochrane CENTRAL, Scopus, and Web of Science for studies reporting on regimens for DR-TB from 1 January 2009 to 8 May 2024. The search terms are summarised in Supplementary Data 1. This study was conducted and reported in accordance with the Preferred Reporting Items for Systematic Reviews and Meta-Analyses statement (PRISMA) and registered in PROSPERO (ID: CRD42024554895).

### Definitions

Mono-resistant TB is defined as a disease caused by *Mycobacterium tuberculosis* strains resistant to only one first-line anti-TB drug (e.g., isoniazid [INH] or rifampicin [RIF]). MDR-TB is defined as a disease caused by *Mycobacterium tuberculosis* strains which are resistant to at least the two core anti-TB drugs, INH and RIF. The WHO definition of XDR-TB has changed over time. It was initially defined as TB caused by MDR-TB strains with additional resistance to any fluoroquinolone (FQ) and at least one of the three injectable second-line drugs (e.g., kanamycin, amikacin, and/or capreomycin). In 2021, the XDR-TB definition was changed based on resistance to Group A MDR-TB drugs (FQs, LZD) and/or BDQ. Similarly, prior to 2021, in clinical practice, pre-XDR-TB was defined as MDR-TB with additional resistance to an FQ or a second-line injectable drug. Subsequently, WHO defined XDR-TB as resistance to an FQ and either LZD or BDQ (e.g. to 2 of the 3 Group A drugs) in 2021. Although the new 2021 pre-XDR-TB definition describes strains with more extensive resistance, only one of the observational studies included in our analysis implemented the new definitions; therefore, the results are pooled in the analysis. A favourable outcome was defined as the cumulative sum of ‘cured’ and ‘treatment completed’, while an unfavourable outcome was defined as the cumulative sum of ‘treatment failure’, ‘lost to follow-up’, and ‘died’.

### Study selection criteria

Eligible studies were required to report treatment outcomes for patients with mono-resistant TB (excluding RIF resistance), RR/MDR-TB, and pre-XDR-TB and/or XDR-TB based on definitions established by the WHO. In studies conducted after 2021, we adhered to the updated WHO definitions, which classify pre-XDR-TB as resistance to RIF, INH, and any FQ, and XDR-TB as resistance to RIF, INH, any FQ, and at least one additional Group A drug. For studies conducted before 2021, we applied the clinical definition of pre-XDR-TB that was used at that time to ensure consistency with clinical practice. Studies were included if they provided sufficient data to categorise the TB strains using these definitions.

We considered both experimental trials (randomised and non-randomised) and observational studies (cohort, case-control, and cross-sectional designs) eligible for inclusion. Case reports, case series, reviews, editorials, conference abstracts, and studies focused solely on children (<14 years) or pregnant women were excluded. Studies were also excluded if they did not report treatment outcomes or if the treatment outcome definitions were inconsistent with the WHO guidelines. Additionally, we only included studies published in English to ensure accessibility for analysis. When duplicate data were identified across multiple publications, we retained the most comprehensive or recent study to avoid redundancy.

### Review process

All identified manuscripts were uploaded to EndNote (Clarivate, London, UK). Duplicates were removed manually. Two reviewers (MA, MCH) independently screened the titles and abstracts of the articles. Subsequently, full texts of all potentially eligible studies were evaluated, and any disagreements were resolved by a third reviewer (MJN) during both the abstract screening and full-text evaluation.

### Data extraction

The extracted data encompassed various parameters, including first author, publication year, study design, period, country and setting, patient demographics: age, sex, body mass index (BMI), risk factors: diabetes mellitus (DM), tobacco usage, HIV, treatment outcome definitions, number of DR-TB cases, treatment duration and treatment outcomes. However, not all extracted data is presented due to word count limitations.

### Quality assessment

The Newcastle-Ottawa Scale (NOS) was used to assess observational studies, while the Cochrane tool (Cochrane, London, UK) was utilised for experimental studies. The NOS scale critically assessed the risk of bias in observational studies across three domains: 1) selection of participants, 2) comparability, and 3) outcomes. Each numbered item within the selection and outcome categories could receive a maximum of one point, while comparability could be assigned a maximum of two points. Scores ranging from 0 to 3, 4 to 6, and 7 to 9 were designated to represent low, moderate, and high-quality studies, respectively.

The Cochrane tool is based on various criteria, including the use of random sequence generation, concealment of allocation to conditions, blinding of participants and personnel, blinding of outcome assessors, completeness of outcome data and other factors, and considerations for selective reporting and other biases. Each study was categorised as at low risk of bias when there was no concern regarding bias, at high risk of bias when there was concern, or at unclear risk of bias if information was absent.

### Meta-analysis

The meta-analysis was performed using Comprehensive Meta-Analysis software, v3.0 (Biostat Inc, Englewood, NJ, USA). The primary outcome of interest was the proportion of patients achieving specific treatment outcomes for the various forms of TB (mono-resistant TB, RR/MDR-TB, pre-XDR-TB, and XDR-TB). Point estimates and corresponding 95% confidence intervals (CIs) were calculated for each included study.

Both random and fixed-effects models were considered to account for variability between studies. The random-effects model was preferred when significant heterogeneity was observed, allowing for variation across studies, while the fixed-effects model was used when the assumption of homogeneity was met. Heterogeneity was assessed using Cochran’s *Q*-test, which determines whether the observed variability in effect sizes is greater than expected by chance. Additionally, the *I*^2^ statistic was used to quantify the percentage of total variation across studies due to heterogeneity rather than chance, with *I*^2^ values greater than 50% indicating substantial heterogeneity.

Publication bias, which could skew the meta-analysis results, was evaluated using Begg’s rank correlation test. This non-parametric test assesses the correlation between study size and effect estimates, with a *P* < 0.05, indicating potential publication bias. In cases of significant bias, appropriate adjustments were made to account for its impact on the overall conclusions.

## RESULTS

### Study characteristics

The Figure shows the flow of the systematic review process, which included 109 studies. Among these, 94 were observational studies, and 15 were experimental studies.^6–114^ As detailed in Table 1 and Supplementary Data 2, the included studies exhibited diverse characteristics regarding DR-TB type, treatment regimen, duration, and treatment outcomes.

**Figure.**
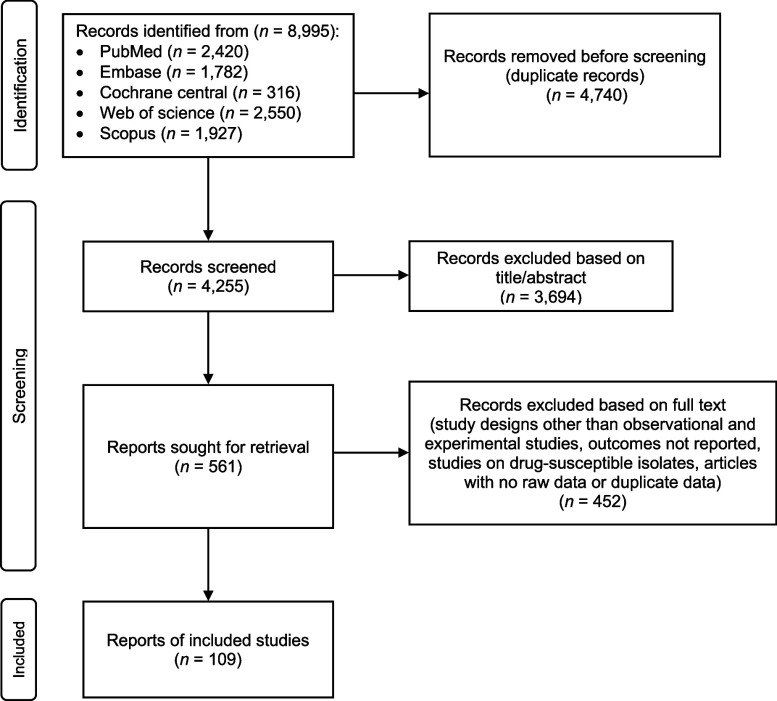
Flow chart of study selection for inclusion in the systematic review and meta-analysis.

**Table 1.** ReDuration of regimens and overall treatment outcomes of included experimental studies.*

Author	Year	DR-TB type	Regimen (follow-up)	Duration	Patients	Treatment success	Failure	Died	LTFU
				Months	*n*	*n*	*n*	*n*	*n*
Shorter regimens									
Goodall (2)^6^	2022	RR	BDQ+Cfz+Z+Lfx/high-dose H+Km	6	134	122	NA	2	2
Nyang’wa^7^ (1)	2022	MDR	BDQ+LZD+Pa+Mfx	6	72	55	0	0	2
Conradie^8^ (1)	2022	Pre-XDR/XDR	BDQ+LZD (1,200 mg, 26 wk)+Pa	6	44	41	0	0	0
Conradie^8^ (2)	2022	Pre-XDR/XDR	BDQ+LZD (1,200 mg, 9 wk)+Pa	6	45	40	0	0	0
Conradie^8^ (4)	2022	Pre-XDR/XDR	BDQ+LZD (600 mg, 26 wk)+Pa	6	45	41	0	0	0
Conradie^8^ (3)	2022	Pre-XDR/XDR	BDQ+LZD (600 mg, 9 wk)+Pa	6	44	37	1	0	1
Overall treatment outcomes with 6-month’ regimens, *n* (%)					384	336 (87.5)	1 (0.3)	2 (0.5)	5 (1.3)
Esmail^9^ (1)	2022	MDR	BDQ+LZD+Lfx+Z+Trd/Eto/high dose H	6–9	49	33	3	4	8
Conradie^10^(1)	2020	MDR	BDQ+LZD +Pa	6–9	107	98	0	6	0
Goodall (1)^6^	2022	RR	Lfx+Cfz+E+Z+BDQ/high dose H+ Pto	9	196	162	NA	3	6
Goodall (3) ^6^	2022	RR	Mfx+Cfz+E+Z(40 wks) /Km+High dose H+Pto	9	187	133	NA	1	3
Mok^11^(1)	2022	MDR	DLM+LZD+ Lfx+Z	9	72	54	5	3	1
Overall treatment outcomes with 6–9-month’ regimens, *n* (%)					611	480 (78.6)	8 (1.3)	17 (2.8)	18 (2.9)
Nunn^12^ (1)	2019	RR	Mfx+Cfz+E+Z +Km+H+Pto	9–11	245	193 (78.8)	NA	8 (2.8)	1 (0.4)
Du^13^ (1)	2019	MDR	Cm+Cfz+Cs+Lfx+Pto+Z	12	67	46 (68.7)	7 (10.4)	2 (3)	12 (17.9)
Longer regimen									
Yao^14^ (1)	2023	MDR	BDQ (6 months)+LZD+Lfx+Cs+Cfz /Lfx+ LZD +Cs+Cfz	18	34	28	5	0	1
Yao^14^ (2)	2023	MDR	BDQ+LZD+Lfx+Cs+Pa/Lfx+ LZD +Cs+Pto	18	34	19	13	1	1
Du^13^ (2)	2019	MDR	Cm+E+Cs+Lfx+Pto+Z	18	68	44	10	1	13
Groote-Bidlingmaier^15^ (1)	2019	MDR/pre-XDR/XDR	DLM + WHO based standard regimen	18–24	224	182	11	9	22
Groote-Bidlingmaier^15^ (2)	2019	MDR/pre-XDR/XDR	Placebo +WHO based standard regimen	18–24	101	85	2	3	11
Nunn^12^ (2)	2019	RR	WHO based standard regimen	20	124	99	NA	3	3
Kang^16^ (1)	2019	MDR	Lfx+Rfb+E+Z+Km/Am/S+Pto+Cs+Pas+LZD	20	77	65	4	2	3
Kang^16^ (2)	2019	MDR	Mfx+Rfb+E+Z+Km/Am/S+Pto+Cs+Pas+LZD	20	74	59	5	0	7
Mok^11^ (2)		MDR	2014 WHO standard regimen	20–24	85	60	11	2	4
Qiujing^17^ (1)	2020	MDR	Z+Am+Lfx+Pto+E	24	45	29	NA	NA	4
Qiujing^17^ (2)	2020	MDR	Z+Am+Mfx+Pto+E	24	45	33	NA	NA	3
Duan^18^ (1)	2019	MDR	Am/Cm+Lfx+Z+E+Pas/Pto+Amx/Clv+Cfz	24	66	43	9	4	10
Duan^18^ (2)	2019	MDR	Am/Cm+Lfx+Z+E+Pas/Pto+Amx/Clv	24	74	35	24	2	9
Tang ^19^ (1)	2014	XDR	LZD+Pto+Z+Mfx/Gfx/Lfx+Pas+Cm/Am+Cfz+Cla	24	33	23	4	2	4
Tang^19^ (2)	2014	XDR	Pa+Z+Mfx/Gfx/Lfx+Pas+Cm/Am+Cfz+Cla	24	32	11	15	3	3
Overall treatment outcomes with ≥18-month’ regimens, *n* (%)					1116	815 (73)	113 (10.1)	29 (2.6)	98 (8.8)
Diacon^20^ (1)	2014	MDR	BDQ +Eto+Z+Ofx+Km+Cs	30	66	38 (57.6%)	NA	10 (12.7)	NA
Diacon^20^ (2)	2014	MDR	Placebo +Eto+Z+Ofx+Km+Cs	30	66	21 (31.8%)	NA	2 (2.5)	NA
Mix regimen									
Esmail^9^ (2)	2023	MDR	WHO-based standard regimen	9–20	44	30	5	4	4
Nyang’wa^7^ (2)	2022	RR	WHO-based standard regimen	9–20	73	34	0	2	2
Overall treatment outcomes with 9–20-month’ regimens, *n* (%)					117	64 (54.7)	5 (4.3)	6 (5.1)	6 (5.1)

* The studies reporting (1) to (4) represent different groups within the same study, where separate analyses were possible based on varying treatment regimens and/or types of drug resistance.

DR-TB = drug-resistant tuberculosis, MDR-TB = multidrug-resistant tuberculosis, XDR-TB = extensively drug-resistant tuberculosis, RR-TB = rifampicin-resistant tuberculosis, Am = amikacin, Cm = capromycin, Lfx = levofloxacin, Z = pyrazinamide, Pto = prothionamide PAS = para aminosalicylic acid, Pa = pretomanid, Amx/Clv = amoxicillin clavulanate, Cfz = clofazimine, LZD = linezolid, BDQ = bedaquiline, Mfx = moxifloxacin, E = ethambutol, H = isoniazid, Km = kanamycin, Gfx = gatifloxacin, CLA = clarithromycin, Cs = cycloserine, Trd = terizidone, Rfb = rifabutin, Ofx = ofloxacin, Eto = ethionamide; NA = not available, LTFU = lost to follow-up.

The observational studies encompassed 41,115 patients with various types of DR-TB, while the experimental studies involved 2,989 DR-TB patients. These studies represented various countries, providing a globally diverse perspective for the current review. Investigated types of DR-TB included INH-R, RR-TB, MDR-TB, pre-XDR-TB, and XDR-TB. Sample sizes varied notably, from 19 patients in a study by Bastard et al. to 3,112 patients in a study by Fan et al. Treatment regimens were heterogeneous, reflecting different national protocols and the evolution of DR-TB treatment strategies. Commonly used regimens comprised combinations of RIF, ethambutol, pyrazinamide, and various FQs, with newer drugs such as BDQ, DLM and Pa.

Treatment durations ranged from 6 to 48 months. The experimental studies (Table 1) showed a range of treatment durations from 6 to 30 months. All study arms reported the regimens’ duration. Out of 32 study arms, 13 (41%) evaluated shorter regimens as follows: six included 6-month regimens, with overall treatment success, failure, death, and loss to follow-up (LTFU) rates of respectively 87.5%, 0.3%, 0.5% and 1.3%; five included regimens lasting 6 to 9 months, with overall treatment success, failure, death and LTFU rates of respectively 78.6%, 1.3%, 2.8% and 2.9%; one with a regimen lasting from 9 to 11 months with 78.8% treatment success, 2.8% death and 0.4% LTFU rates; and one with a 12-month regimen, with 68.7% treatment success, 10.4% treatment failure, 2.3% death and 17.9% LTFU rates.

Longer regimens lasting from 18 to 24 months were evaluated in 17 study arms (53%), and treatment success was available for 15 study arms with overall treatment success, failure, death and LTFU rates of respectively 73%, 10.1%, 2.6% and 8.8%. Two (6%) study arms included regimens of mixed duration (9 ∼20 months) with overall treatment success, failure, death and LTFU rates of respectively 54.7%, 4.3%, 5.1% and 5.1%. Out of 122 observational study arms (Supplementary Data 2), 15 (12.3%) had no details on treatment duration. Of the remaining 107, 37 (34.6%) investigated shorter regimens.

Six-month regimens (*n* = 10) achieved overall treatment success, failure, death and LTFU rates of respectively 81.1%, 2.5%, 5.5% and 6.6%, while 6–12-month regimens (*n* = 27) achieved overall treatment success, failure, death and LTFU rates of respectively 75.5%, 4.3%, 10.8% and 6.2%. Two arms of a single study evaluated a 13-month regimen, with overall treatment success, failure, death and LTFU rates of respectively 46%, 9.8%, 23.4% and 20.9%.

The longer (14–48 months) regimens were evaluated by 67 studies or arms (62.6%) with overall treatment success, failure, death and LTFU rates of respectively 50.1%, 7.1%, 7.4% and 10.2%. One study included a mixture of shorter and longer regimens, achieving 25.9% treatment success, 8.9% treatment failure, 51.8% death and 9.8% LTFU rates.

Overall, the data demonstrated a higher proportion of studies with the 18- to 24-month duration, with a clear prominence of 24-month-long regimens. Due to the heterogeneity among studies, it was not possible to describe specific trends related to regimen use, regimen composition, specific drug use and duration. The mean age of participants across observational studies was 40.4 years, whereas in the experimental studies, it was 39.3 years.

The proportion of male patients was consistently higher across studies, with some studies reporting as high as 94% male participation (range: 37–94). HIV co-infection was notable in certain studies, particularly those conducted in regions with high HIV prevalence. For instance, Ndjeka et al. reported high rates of HIV co-infection among their study populations in South Africa, with 493 out of 688 patients being HIV co-infected in one cohort. Twenty-one studies included the prevalence of hepatitis C co-infection (not presented due to incompleteness of data reported). BMI data were inconsistently reported, ranging from underweight to normal weight. Prevalent diabetes and smoking history were less frequently reported. For example, Velayutham et al. noted that 1,417 out of 11,519 participants had diabetes, and 1,476 were smokers.

### Quality assessment

The quality assessment of observational studies, evaluated using the Newcastle-Ottawa Scale (NOS) tool, was conducted on 94 studies (Supplementary Data 3). All studies demonstrated moderate to high methodological quality, with scores ranging from 4 to 7 out of a maximum of 9, indicating robust methodology across various domains such as representativeness of the exposed cohort, selection of the non-exposed cohort, ascertainment of exposure, demonstration that the outcome of interest was not present at the start of the study, adjustment for the most important risk factors, adjustment for other risk factors, assessment of outcome, follow-up length, and loss to follow-up rate. Limitations were observed across studies in comparability and adjustment for other risk factors, which could affect the reliability and validity of findings. Given the non-experimental structure of all observational studies and the clinical context of tuberculosis treatment, all observational studies were penalised for not selecting non-exposed cohorts.

The quality assessment of experimental studies, conducted using the Cochrane tool, revealed variations in methodological rigour among the included trials (Supplementary Data 4). Among the total 15 studies assessed, the majority exhibited low risk across all domains, indicating that the methodology was robust. Specifically, 3 out of 15 studies (Diacon et al.,^20^ Conradie et al.,^8^ Groote-Bidlingmaier et al.^15^) showed low risk across all domains. Additionally, two studies (Qiujing et al.,^17^ Conradie et al.^10^) showed high risk in random sequence generation, allocation concealment, blinding of participants and personnel, and blinding of outcome assessment.

### Pooled treatment outcomes in observational studies

Supplementary Data 5 summarises the results of pooled treatment outcomes based on the type of DR-TB and lists the various treatment regimens utilised in observational studies. For mono-resistance, the overall favourable outcome rate was 82.9%, ranging from 78.7% to 87.2% (Supplementary Data 5,Supplementary Data 6), while the unfavourable outcome rate was 15.8%, varying from 13.3% to 18.4%. As shown in Supplementary Data 7, no evidence of publication bias was observed. When treatment regimens of mono-resistant TB included FQ, the favourable outcome rate increased to 84.7%, ranging from 79.1% to 90.3%. However, without FQ, the favourable outcome rate decreased to 81.1%, ranging from 72.7% to 89.6%. As shown in Supplementary Data 8, rates of favourable outcomes generally show an increasing trend over time, starting from 69.4% in 2012 and reaching up to 84.9% in 2022.

For RR/MDR-TB, the overall favourable outcome rate was 68.4% (Supplementary Data 5,Supplementary Data 9), varying from 64.6% to 72.3%, with no evidence of publication bias (Supplementary Data 9, Supplementary Data 10). The unfavourable outcome rate was 29.0%, ranging from 25.3% to 32.7%. As indicated in Supplementary Data 11, there was a general increasing trend in favourable outcomes from 2011 to 2024. Early studies (e.g., Farley et al. with an effect size of 40.07) showed smaller effect sizes than more recent studies (e.g., Zhang et al., with an effect size of 68.18).

For pre-XDR/XDR-TB, the overall favourable outcome rate was 54.4% (ranging from 42.3% to 66.5%) (Supplementary Data 5, Supplementary Data 12), with no evidence of publication bias (Supplementary Data 12, Supplementary Data 13). The unfavourable outcome rate was 43.9% (32.5% to 55.4%). Supplementary Data 14 shows a clear increase in favourable outcomes over time. Early studies, such as Tang et al., reported lower effect sizes (13.02), whereas more recent studies, such as Nguyen et al., reported higher effect sizes (52.85).

For studies with a mix of MDR/pre-XDR/XDR-TB, the overall favourable outcome rate was 70.6% (range: 67.4–73.8), with no evidence of publication bias (Supplementary Data 15, Supplementary Data 16). The unfavourable outcome rate was 27.1% (range: 23.8% to 30.4%). The overall trend in favourable outcomes (Supplementary Data 17) indicates an increase from 2010 to 2024.

Table 2 shows that favourable outcomes were reported from different WHO regions. In the African region, the favourable outcome rates were 65.0% (range: 57.66–72.39) for RR/MDR TB, 40.0% (range: 13.78–66.26) for pre-XDR/XDR TB, and 63.4% (range: 53.08–73.74) for MDR/pre-XDR/XDR-TB. The favourable outcome rate for mono-resistant TB in the American region was 79.5% (range: 68.97–90.00). In the European region, the favourable outcome rates were 70.5% (range: 63.05–78.03) for RR/MDR-TB, 64.7% (range: 40.29–89.17) for pre-XDR/XDR-TB, and 65.6% (range: 60.45–70.64) for MDR/pre-XDR/XDR-TB. The favourable outcome rate for RR/MDR-TB in the South-East Asia Region was 67.4% (range: 59.75–75.09). In the Western Pacific Region, the favourable outcome rates were 85.6% (range: 78.98–92.20) for mono-resistant TB, 70.2% (range: 62.19–78.18) for RR/MDR TB, 59.9% (range: 43.75–76.12) for pre-XDR/XDR-TB, and 78.5% (range: 76.01–80.95) for MDR/pre-XDR/XDR-TB. The favourable outcome rate for MDR/pre-XDR/XDR TB in the Eastern Mediterranean Region was 69.0%.

**Table 2.** Summary of results based on WHO regions (observational studies).

Region	*n*	Favorable outcome	Unfavorable outcome	Failure	Died	LTFU
		% (95% CI)	% (95% CI)	% (95% CI)	% (95% CI)	% (95% CI)
African						
RR/MDR	3,758	65.02 (57.66 to 72.39)	35.25 (27.38 to 43.11)	12.06 (–5.83 to 29.95)	10.46 (5.78 to 15.15)	13.07 (8.35 to 17.79)
Pre-XDR/XDR	487	40.02 (13.78 to 66.26)	56.51 (31.97 to 81.05)	22.61 (–16.80 to 62.03)	21.32 (8.34 to 34.30)	19.03 (5.89 to 32.18)
MDR/pre-XDR/XDR	871	63.41 (53.08 to 73.74)	36.59 (25.48 to 47.70)	4.11 (0.40 to 7.82)	21.01 (11.83 to 30.18)	11.97 (8.01 to 15.93)
Americas						
Mono	309	79.48 (68.97 to 90.00)	16.58 (-1.66 to 34.82)	1.78 (0.66 to 2.90)	7.30 (0.66 to 13.95)	15.29 (14.73 to 15.86)
Europe						
RR/MDR	2978	70.54 (63.05 to 78.03)	27.85 (19.21 to 36.50)	10.19 (4.91 to 15.48)	10.41 (4.53 to 16.28)	12.06 (9.56 to 14.56)
Pre-XDR/XDR	381	64.73 (40.29 to 89.17)	34.43 (17.41 to 51.44)	31.62 (-3.81 to 67.06)	18.36 (5.16 to 31.55)	7.77 (4.68 to 10.87)
MDR/pre-XDR/XDR	3470	67.27 (61.45 to 73.09)	25.07 (19.70 to 31.70)	8.69 (6.70 to 10.67)	7.94 (2.82 to 13.06)	13.69 (11.03 to 16.34)
South-East Asia						
RR/MDR	2184	67.42 (59.75 to 75.09)	30.41 (20.88 to 39.93)	3.18 (–0.20 to 6.55)	11.64 (9.05 to 14.23)	16.31 (7.87 to 24.75)
Western Pacific						
Mono	925	85.59 (78.98 to 92.20)	15.59 (9.31 to 21.87)	6.91 (–1.24 to 15.05)	8.15 (3.46 to 12.83)	4.87 (–1.10 to 10.84)
RR/MDR	5069	70.19 (62.19 to 78.18)	24.89 (18.42 to 31.35)	12.03 (6.23 to 17.84)	4.38 (1.55 to 7.20)	11.04 (6.34 to 15.73)
Pre-XDR/XDR	109	59.94 (43.75 to 76.12)	40.08 (14.82 to 65.34)	2.07 (0.65 to 3.48)	14.92 (1.10 to 28.75)	23.77 (10.02 to 37.52)
MDR/pre-XDR/XDR	6898	78.48 (76.01 to 80.95)	21.37 (18.70 to 24.05)	6.89 (3.66 to 10.12)	5.76 (3.59 to 7.93)	7.98 (4.57 to 11.38)
Eastern Mediterranean						
MDR/pre-XDR/XDR	271	69.00	30.00	1.00	17.50	12.50

RR = rifampicin-resistant; MDR = multidrug-resistant; XDR = extensively drug-resistant; LTFU = lost to follow-up; CI = confidence interval.

### Pooled treatment outcomes in experimental studies

Table 3 summarises results from experimental studies based on different types of DR-TB. For RR/MDR-TB, the favourable outcome rate was 69.6% (range: 61.02–87.20) with no evidence of publication bias (Supplementary Data 18, Supplementary Data 19). The unfavourable outcome rate was 22.9% (range: 17.25–28.37). The overall trend in Supplementary Data 20 shows no clear patterns in favourable outcome rates across the studies from 2019 to 2023.

**Table 3.** Summary of results based on type of DR-TB (experimental studies).

DR-TB	Favorable outcome	Unfavorable outcome	Failure	Died	LTFU
	% (95% CI)	% (95% CI)	% (95% CI)	% (95% CI)	% (95% CI)
RR/MDR	69.61 (61.02–87.20)	22.92 (17.25–28.37)	10.04 (6.10–13.17)	5.77 (2.73–9.21)	7.62 (3.68–11.14)
Pre-XDR/XDR	79.22 (60.56–97.87)	20.79 (2.82–39.87)	12.12 (11.08–13.72)	7.33 (4.30–9.79)	12.00 (11.08–13.72)
MDR/pre-XDR/XDR	85.82 (75.45–96.19)	13.75 (4.21–23.63)	4.87 (4.27–5.47)	3.98 (3.32–4.94)	9.73 (9.31–10.16)

RR = rifampicin-resistant; MDR = multidrug-resistant; XDR = extensively drug-resistant; DR-TB = drug-resistant tuberculosis, LTFU = lost to follow-up; CI = confidence interval.

For pre-XDR/XDR-TB, the favourable outcome rate was 79.2% (range: 60.56–97.87) and for RR/MDR/pre-XDR/XDR-TB, the favourable outcome rate was 85.8% (range: 75.45–96.19).

## DISCUSSION

The aim of the present study was to perform an SLR and meta-analysis to describe the evolution of treatment outcomes in DR-TB with a focus on efficacy and effectiveness. Over the past 15 years, the WHO issued several major revisions and updates to the guidelines for the management of TB and RR/MDR-TB, endorsing a range of new technologies such as rapid diagnostics tests to improve case detection and recommending the inclusion of later-generation FQs in the regimens as well as the routine use of repurposed orally administered drugs such as LZD or clofazimine (CFZ) in the treatment of DR-TB. The updated treatment guidelines recommend the inclusion of CFZ as a core component of DR-TB regimens, highlighting its efficacy and manageable safety profile in combination with other Group A drugs such as FQs and BDQ.^115^

Another important change in the management of TB was the recommendation of all-oral as well as shorter regimens for DR-TB. The WHO currently recommends two shorter regimens for RR/MDR-TB―a fixed 6-month regimen and a 9∼12-month regimen for eligible patients in addition to the longer regimens of 18–20 months.^3,4^ Furthermore, according to the 2023 update of The Working Group on New TB Drugs, which tracks the global TB pipeline, there are more than 19 promising compounds in different stages of clinical development, as well as various combinations of existing, repurposed, and new agents, currently investigated in phase 2 or 3 clinical trials as components of potential new treatment regimens.^116^

The current analysis of treatment outcomes, performed separately for observational and experimental studies, demonstrated a gradual increase in treatment success of DR-TB over the period 2009 to 2024. It was possible to study treatment outcomes within observational studies as the number of studies was sufficiently high. The overall treatment success rate for mono-resistant TB was 82.9%, increasing over time (from 69.4% in 2012 to 84.9% in 2022).

The large intra-study variability on the rates of loss to follow-up (LTFU) (5.7% to 12.1%), reflecting the performances in different settings and over time, must be considered when evaluating the results. Further research is needed to assess the role of improvement in clinical management vis-a-vis advances in people-centred care and programmatic improvement in reducing the LTFU rates.

In this review, the overall treatment success rates in observational studies for RR/MDR-TB and pre-XDR-TB /XDR-TB were 68.4% and 54.4%, respectively, with an increasing trend over time. The treatment success rates for pre-XDR and XDR-TB were much lower, with a large range (43.32–66.48). For both RR/MDR-TB and pre-XDR/XDR-TB, the wide range observed for each individual outcome analysed reflected the difference in study settings, as observational studies tend to reflect programmatic results, which are very different from those of controlled clinical trials. It is important to mention that the studies between 2011 and 2024 reporting a mix of MDR- and pre-XDR-TB cases showed a pooled treatment success of 70.6%, which can be due to various reasons, including wider availability of treatment options.

The analysis of the outcomes emerging from experimental studies, lower in number, demonstrated 69.9% treatment success for RR/MDR-TB, consistent with the results from observational studies (with a modest increase for the period 2019–2023) and a much higher value for pre-XDR/XDR-TB (79.2%), and for studies including a mix of patients from the previous two groups (85.8%). These results are possibly explained by factors such as stricter patient monitoring, the availability of shorter regimens, and differences in study design. All but one of the studies published after 2020 included BDQ (7 studies), DLM (1 study), or Pa (5 studies).

There was also geographic variation observed across studies. For example, more studies were conducted in Asian countries, particularly China and South Korea. China provided the highest number of studies, with 14 studies (10 cohorts and 4 experimental). South Korea also had a notable presence, with 9 studies (7 cohorts and 2 experimental studies). India was another major contributor, with 7 cohort studies. In terms of observational studies, the top contributing countries were China (10 studies), South Africa (9), South Korea and India (7). The highest number of experimental studies was reported by China (4), while four studies were conducted in multiple countries. There is a noticeable lack of studies from Latin American countries, with a study from Peru.

Despite the progress made in recent years resulting in a steady improvement of global treatment success rates, from 50% in 2012 to 63% in the 2020 cohort (ranging from 55% to 73%, depending on the region),^1^ the rates are still lower than the success rates of 85% for drug-susceptible TB and far below the WHO targets of 75%. In addition, the 2020 milestones of the End TB Strategy (35% mortality reduction and 20% incidence reduction) have not been met, indicating that areas for further improvement remain.^117^

While we have described improvement in outcomes over time in all categories and both observational and experimental studies, there appears to be a ‘ceiling effect’. The treatment success of mono-resistant cases reached 80–85% (beginning around 2015), 70% for RR/MDR-TB cases (beginning around 2019), and 70 to 80% for pre-XDR/XDR-TB in observational and experimental studies, respectively (beginning around 2019). While for combined RR/MDR and XDR-TB cases, it was 70 and 85%, respectively (beginning around 2019). The use of FQs with the addition of LZD before and BDQ, DLM or Pa afterwards, often within innovative and shortened regimens, allowed us to reach this important improvement in efficacy.

At least 4 of the 15 experimental studies included in this review were influential in WHO guidelines. A shorter all-oral BDQ-containing regimen of 9–12 months was recommended in 2020 WHO guidelines, based on published results from the study by Nunn et al. In addition, the evidence generated by two randomised clinical trials, the TB-PRACTECAL and ZENIX, led the WHO to recommend the 6-month BPAL(M) regimen over the longer regimens in 2022 guidelines. However, there is evidence of increasing resistance rates to WHO Group A drugs, in particular to FQS and BDQ.

The prevalence of resistance to FQs has ranged from 0.2% to 3.6% among newly diagnosed patients.^118–122^ It has been shown that the use of FQs for more than 10 days and more than 60 days before TB diagnosis is associated with a high risk of FQ-resistant TB.^123^ On the other hand, a systematic review^124^ demonstrated that the frequency of phenotypic-acquired BDQ resistance was 2.2% and of genotypic-acquired BDQ resistance was 4.4%. However, Chesov et al. reported that acquired BDQ resistance can occur in >15% of MDR-TB patients. An insufficient number of effective drugs, baseline FQ resistance, and previous exposure to CFZ were associated with acquired BDQ resistance. Currently, drug susceptibility testing (DST) for WHO group A drugs beyond FQs is challenging due to programmatic reasons as well as differences in microbiological standards. DST for new medicines is to be developed as early as possible so that the new treatment regimen can be as efficient as possible and prevent further drug resistance.^125–127^

Our analysis is subject to limitations, including the inability to conduct a regimen-level analysis given the heterogeneity and scope of this review. The heterogeneity of studies also prevented stratification of DR-TB treatment regimens across socioeconomic determinants, duration of treatment, or risk factors. Limiting to literature published in English was an additional limitation. Many experimental and observational studies included in this review were conducted during the COVID-19 pandemic, with challenges in recruitment, retention and/or follow-up of patients; hence, these results might be interpreted with caution when compared with studies conducted before the pandemic. Future analysis should aim to describe and synthesise safety and tolerability outcomes to complement the current analysis of treatment outcomes. While it was not possible to access the individual patient data (IPD) of the cases analysed in our study, future analysis should aim to conduct a more detailed analysis based on individual data enabled by the recent launch of the UCL/WHO IPD platform.

The current SLR and meta-analysis demonstrated a trend of steady improvement in treatment outcomes for DR-TB after a long period of stagnation. However, current regimens appear to have reached a ceiling effect, and new drugs/regimens are needed to maintain or further improve efficacy/ effectiveness and address issues of emerging resistance to cornerstone components of the current go-to regimens.

## Supplementary Material


