# Supplementary material for: 15-year trends in efficacy and effectiveness of treatment outcomes in drug-resistant pulmonary TB
Source: IJTLD Open. 2025 Apr 9;2(4):187–98. doi: 10.5588/ijtldopen.24.0620 (PMC11984524; doi:10.5588/ijtldopen.24.0620)
Supplement: Supplementary file 1 [file ijtldopen24-0620_supplementarydata1.docx]

**15-year trends in efficacy and effectiveness of treatment outcomes in drug-resistant pulmonary TB**

**Supplementary Data 1. Summary of the search terms used**

*Cochrane CENTRAL*

“Tuberculosis” OR "mycobacterium tuberculosis" OR “TB” OR “MTB” OR "tuberculosis":ti,ab,kw OR "Multi-drug resistant" OR "multi drug resistant" OR "multi drug-resistant" OR “multidrug resistant” OR "multi-drug resistance" OR "multi drug resistance" OR "multi drug-resistance" OR “multidrug resistance” OR "MDR" OR “MDR-TB”:ti,ab,kw OR "extensively drug resistant" OR "extensively drug-resistant" OR "extensively drug resistance" OR "extensively drug-resistance" OR “extensive drug resistant” OR “extensive-drug resistant” OR “extensive drug-resistant” OR “XDR” OR “XDR-TB” OR “Pre-XDR” OR “Pre-XDR-TB” OR “pre-XDR TB” OR “Rifampicin Resistant” OR “RR”:ti,ab,kw AND "outcome":ti,ab,kw AND NOT ("systematic review" OR "meta-analysis" OR "case report" OR "case series" OR "editorial" OR "letter")

*Scopus*

(TITLE-ABS-KEY("Tuberculosis" OR "mycobacterium tuberculosis" OR "TB" OR "MTB" )) AND (TITLE-ABS-KEY("Outcome*")) AND (TITLE-ABS-KEY("Multi-drug resistant" OR "multi drug resistant" OR "multi drug-resistant" OR "multidrug resistant" OR "multi-drug resistance" OR "multi drug resistance" OR "multi drug-resistance" OR "multidrug resistance" OR "MDR" OR "MDR-TB" OR "extensively-drug resistant" OR "extensively drug resistant" OR "extensively drug-resistant" OR "extensively-drug resistance" OR "extensively drug resistance" OR "extensively drug-resistance" OR "extensive drug resistant" OR "extensive-drug resistant" OR "extensive drug-resistant" OR "XDR" OR "XDR-TB" OR "pre-XDR-TB" OR "pre-XDR TB" OR "pre-XDR" OR "DR TB" OR "DR-TB" OR "drug resistance" OR "drug-resistance" OR "drug resistant" OR "drug-resistant" )) AND NOT ("systematic review" OR "meta-analysis" OR "case report" OR "case series" OR "editorial" OR "letter")

*Web of Science*

TS= (Tuberculosis OR “mycobacterium tuberculosis” OR “TB” OR “MTB”)

TS= ((” outcome*”))

TS=(("Multi-drug resistant" OR "multi drug resistant" OR "multi drug-resistant" OR “multidrug resistant” OR "multi-drug resistance" OR "multi drug resistance" OR "multi drug-resistance" OR “multidrug resistance” OR "MDR" OR “MDR-TB” OR "extensively-drug resistant” OR ”extensively drug resistant” OR ”extensively drug-resistant” OR ”extensively-drug resistance” OR ”extensively drug resistance” OR ”extensively drug-resistance” OR ”extensive drug resistant” OR ”extensive-drug resistant” OR ”extensive drug-resistant” OR ”XDR” OR “XDR-TB” OR “pre-XDR-TB” OR “pre-XDR TB” OR “pre-XDR” OR ”DR TB” OR ”DR-TB” OR ”drug resistance” OR ”drug-resistance” OR ”drug resistant” OR “drug-resistant”) )

#1 AND #2 AND #3 NOT TS=” systematic review” NOT TS= “meta-analysis” NOT TS= “case report” NOT TS= “case series” NOT TS=” editorial” NOT TS=” letter”

Filters applied: Year 2009 and onwards

**Supplementary Data 2. Characteristics of included cohort studies. The studies reporting (1) to (3) are those in which it was possible to analyse separately the different arms/regimens.**

| **Author** | **Year** | **DR-TB type** | **Regimen (Follow-up)** | **Duration** | **N. of patients** | **Treatment success** | **Failure** | **Died** | **LTFU** |
| --- | --- | --- | --- | --- | --- | --- | --- | --- | --- |
| **SHORTER REGIMEN** | | | | | | | | | |
| Kwon (1) ^21^ | 2023 | MDR/ Pre-XDR/XDR | BDQ + DLM + LZD + Cfz + Lfx/Mfx + Km + Am + Pto + Cs + Pas + high dose H | 6 months | 754 | 594 | 31 | 79 | 37 |
| Kwon (2) ^21^ | 2023 | MDR/ Pre-XDR/XDR | BDQ + DLM + LZD + Cfz + Lfx/Mfx + Km + Am + Pto + Cs + Pas | 6 months | 31 | 26 | 0 | 3 | 1 |
| Velayutham^22^ | 2022 | INHR | Lfx/Mfx + Rif + E + Z | 6 months | 11519 | 9440 | 252 | 579 | 728 |
| Kwon ^23^ | 2021 | MDR/XDR | BDQ + LZD + Cfz + Mem/Clv+Cyc | 6 months | 28 | 23 | 2 | 1 | 2 |
| Auchynka ^24^ | 2021 | MDR/XDR | BDQ + DLM + LZD + Mfx + Lfx + + Cfz + Cs + Trd + Z + Imp + Am + Cm + Pto + Eto + Amx/Clv | 6 months | 125 | 110 | 5 | 3 | 7 |
| Ndjeka ^25^ | 2018 | Pre-XDR/XDR | BDQ + LZD + Cfz+Z+E+high dose H+ Pas+Cm+Km+Lfx+Eto+Trd | 6 months | 200 | 146 | 9 | 25 | 20 |
| Kim ^26^ | 2017 | INHR | Inh+E+Z+FQs+R | 6 months | 56 | 56 | 0 | NM | 0 |
| Lew (1) ^27^ | 2016 | INHR | H+Rif+Z+E +H+Rif | 6 months | 288 | 247 | 11 | 6 | 22 |
| Chien ^28^ | 2014 | INHR | H+Rif+E+Z+STR+FQs/ H+Rif+E+Z+FQs | 6 months | 395 | 328 | 11 | 56 | NM |
| Gegia ^29^ | 2012 | INHR | WHO based standard regimen | 6 months | 909 | 631 | 37 | 30 | 128 |
| **Overall Treatment outcomes with 6 months regimens** | | | | | **14305** | **11601 (81.1%)** | **358 (2.5%)** | **782 (5.5%)** | **945 (6.6%)** |
| Padmapriyadarsini ^30^ | 2022 | MDR | BDQ + DLM + LZD + Mfx + Cfz + Pto | 6-8 months | 165 | 139 | NM | 4 | NM |
| Lee (2) ^31^ | 2016 | INHR | R+E+Z | 7 months | 65 | 55 | 6 | NM | NM |
| Mpagama ^32^ | 2013 | MDR | WHO based standard regimen | 7 months | 61 | 13 | 41 | 4 | 3 |
| Lew (2) ^27^ | 2016 | INHR | H+Rif+Z+E+STR +H+Rif+Z+E +H+Rif+E | 8 months | 138 | 75 | 29 | 8 | 19 |
| Avaliani ^33^ | 2021 | RR/MDR | BDQ + LZD + Lfx + Cfz + Cs | 9 months | 25 | 22 | 0 | 1 | 2 |
| Bastard ^34^ | 2019 | MDR | Z+H+E+Mfx+Km+Pto+Cfz /Z+E+Mfx+Pto+Cfz | 9 months | 19 | 16 | 0 | 0 | 0 |
| Lee (1) ^31^ | 2016 | INHR | R+E+Z+S+FQs | 9 months | 75 | 73 | 1 | NM | NM |
| Lotz (1) ^35^ | 2023 | RR/MDR | WHO based standard regimen | 9-11 months | 105 | 72 | 1 | 23 | 9 |
| Nguyen ^36^ | 2022 | RR | BDQ + LZD + Mfx + Cfz + Cs + Z | 9-11 months | 106 | 95 | 4 | 1 | 6 |
| Kumari ^37^ | 2022 | RR | WHO based standard regimen | 9-11 months | 360 | 303 | 2 | 33 | 22 |
| Kumar G ^38^ | 2022 | MDR | Mfx + Km/Am +Eto +Cfz + Z+ High dose H + E | 9-11 months | 135 | 85 | 3 | 24 | 7 |
| Indarti (1) ^39^ | 2022 | MDR | Mfx + Km/Am +Eto +Cfz + Z+ High dose H + E | 9-11 months | 65 | 23 | 2 | 6 | 32 |
| Schwoebel^40^ | 2020 | RR | Km + Cfz + Mfx + E +high dose H + Z + Pto /Mfx + E + Z + Cfz | 9-11 months | 1006 | 823 | 60 | 79 | NM |
| Anh ^41^ | 2020 | RR/MDR | Km + Lfx + Cfz + E + Z + H + Pto | 9-11months | 302 | 259 | 16 | 13 | 14 |
| Ndjeka (1) ^42^ | 2022 | RR | BDQ + Mfx + Cfz | 9–12 months | 688 | 507 | 5 | 117 | 59 |
| Ndjeka (2) ^42^ | 2022 | RR | BDQ + Mfx + Cfz + Am + Cm + S + Km | 9–12 months | 699 | 421 | 14 | 159 | 105 |
| Koirala ^43^ | 2021 | RR/MDR | WHO based standard regimen | 9–12 months | 301 | 239 | 8 | 36 | 16 |
| Huerga^44^ | 2022 | RR/MDR | LZD + Cfz + Z + Mfx + Cs + Eto/Pto + Am + Pas | 10 months | 302 | 239 | 21 | 23 | 19 |
| Sidamo (1) ^45^ | 2021 | MDR | BDQ + DLM + LZD + Mfx + Cfz + Pa+ H + E + Z + Cs | 10 months | 43 | 22 | 7 | 5 | 9 |
| Schechter ^46^ | 2017 | INHR | Rif+Z+E+FQ | 10 months | 59 | NM | NM | 8 | 0 |
| Wang (1) ^47^ | 2014 | INHR | E+H+R+Z | 10 months | 44 | 36 | 2 | 4 | 1 |
| Wang (2) ^47^ | 2014 | INHR | E+H+R+Z | 10 months | 90 | 78 | 2 | 7 | 2 |
| Romanowski^48^ | 2017 | INHR | RIF + E + Z | 10.5 months | 165 | 140 | 2 | 6 | NM |
| Aslan^49^ | 2023 | INHR/MDR | Lfx/Mfx + E + Z + R + H | 12 months | 188 | 170 | 5 | 9 | NM |
| Kang (1) ^50^ | 2020 | MDR/ Pre-XDR/XDR | BDQ + LZD + Km + Lfx + Cyc | 12 months | 107 | 86 | 1 | 12 | 8 |
| Kang (3) ^50^ | 2020 | MDR/ Pre-XDR/XDR | LZD + Am/Km + Lfx + Cyc | 12 months | 67 | 58 | 3 | 3 | 3 |
| Kang (2) ^50^ | 2020 | MDR/ Pre-XDR/XDR | DLM + LZD + Km + Lfx + Cyc | 12 months | 108 | 95 | 1 | 8 | 4 |
| **Overall Treatment outcomes with 6-12 months regimens** | | | | | **5488** | **4144 (75.5%)** | **236 (4.3%)** | **593 (10 .8%)** | **340 (6.2%)** |
| Farley (1) ^51^ | 2011 | MDR | WHO based standard regimen (pts with HIV infection) | 13 months | 287 | 115 | 12 | 101 | 59 |
| Farley (2) ^51^ | 2011 | MDR | WHO based standard regimen (pts HIV negative) | 13 months | 470 | 233 | 62 | 76 | 99 |
| **Overall Treatment outcomes 13 months regimens** | | | | | **757** | **348 (46%)** | **74 (9.8%)** | **177 (23 .4%)** | **158 (20 .9%)** |
| **LONGER REGIMEN** | | | | | | | | | |
| Nkurunziza (1) ^52^ | 2018 | XDR | LZD + Cm + Km + Am + Mfx + Ofx + Lfx + Z + E + Pas + Cfz + Eto + Trd + + Azm + High dose H + Amx/Clv + Clr | 14 months | 53 | 11 | 7 | 14 | 12 |
| Bonnet ^53^ | 2015 | MDR/ Pre-XDR/XDR | E+Z+Ofx/Cfx+Lfx/Mfx+Am/Km+Cm | 15–24 months | 1433 | 808 | NN | 127 | 333 |
| Mikiashvili^54^ | 2024 | MDR/ Pre-XDR/XDR | BDQ + DLM + LZD + Cfz + Lfx/Mfx | 16 months | 106 | 69 | 8 | 4 | 25 |
| Sidamo (2) ^45^ | 2021 | MDR | BDQ + DLM + LZD + Mfx + Cfz + Pto + Cs | 17 months | 37 | 30 | 2 | 3 | 2 |
| Prince ^55^ | 2023 | MDR | BDQ + LZD + Lfx/Mfx + Cm + Cs | 18 months | 62 | 52 | 2 | 4 | 4 |
| Massud* ^56^ | 2023 | RR/MDR/XDR | WHO based standard regimen | 18 months | 271 | 187 | 2 | 48 | 34 |
| Sun ^57^ | 2021 | MDR | Am + Mfx + Cs + Pto + Pa + Z / Mfx + Cs + Pto + Z | 18 months | 114 | 91 | 10 | 2 | 10 |
| Barvaliya ^58^ | 2020 | Pre-XDR/XDR | BDQ + LZD + Km/Cm + Lfx/Mfx + Eto + Cs + Pas+ Cfz+ H/Clr + Z | 18 months | 127 | 102 | 10 | 14 | NM |
| Riccardi ^59^ | 2019 | MDR/Pre-XDR | LZD + Mfx + Am + Cfz + Mer | 18 months | 134 | NM | NM | NM | NM |
| Nkurunziza (3) ^52^ | 2018 | Pre-XDR | LZD + Cm + Am + Mfx + Lfx + Z + E + Pas + Cfz + Eto + Trd + Azm + High dose H + Amx/Clv + Clr | 18 months | 7 | 4 | 1 | 1 | 1 |
| Hoa (1) ^60^ | 2014 | MDR | WHO based standard regimen | 18 months | 79 | 67 | 2 | 3 | 5 |
| Hoa (2) ^60^ | 2014 | MDR | Am/Km+Cm+Flq+Ofx/Lfx/Mfx/Gfx/Cip+Eto+Cs+Pas | 18 months | 203 | 109 | 5 | 0 | 52 |
| Bendayan ^61^ | 2010 | MDR/XDR | WHO based standard regimen | 18 months | 132 | 70 | 13 | 40 | 9 |
| Fan ^62^ | 2023 | RR/ MDR/ Pre-XDR/XDR | BDQ + DLM + LZD + Cfz + Lfx/Mfx + Am + Pto + Cs + Z + E + Am | 18-20 months | 3112 | 45 | 1 | 3 | 8 |
| Zhang (1) ^63^ | 2022 | RR/MDR/XDR | BDQ + LZD + Cfz + Lfx/Mfx + Am + Pto + Cs + Z + E + Pas | 18–20 months | 102 | 94 | 3 | 1 | 4 |
| Zhang (2) ^63^ | 2022 | RR/MDR/XDR | LZD + Cfz + Lfx/Mfx + Am + Pto + Cs + Z + E + Pas | 18–20 months | 100 | 63 | 27 | 1 | 9 |
| Velásquez ^64^ | 2014 | MDR/XDR | WHO based standard regimen | 18–23 months | 614 | 406 | 54 | 30 | 123 |
| Indarti (2) ^39^ | 2022 | MDR | BDQ +Cs + Z + H +Pas | 18-24 months | 34 | 18 | 1 | 4 | 10 |
| Burham^65^ | 2022 | MDR | Km/Am +Cs + Eto + Lfx + Z + E | 18-24 months | 409 | 252 | 8 | 44 | 105 |
| Oelofse (1) ^66^ | 2021 | MDR/XDR | BDQ + LZD + Ptm | 18-24 months | 109 | 98 | 2 | 7 | 1 |
| Oelofse (2) ^66^ | 2021 | MDR/XDR | Z + Cfz + Lfx + Pas + Trd | 18-24 months | 102 | 66 | 5 | 19 | 11 |
| Lee ^67^ | 2017 | MDR | DLM + LZD + Rfb + E + Z + Mfx/Lfx + Km/Am/Cm + Pto + Cs + Pas + Lzd + DLM + High dose H | 18-24 months | 208 | 177 | 10 | 11 | 1 |
| Jeong ^68^ | 2015 | MDR/XDR | Rfb+E+Z+Am/Km+FQ+Pto | 18-24 months | 337 | 272 | 15 | 5 | 23 |
| Rodriguez ^69^ | 2012 | MDR | WHO based standard regimen | 18-24 months | 289 | 108 | 13 | 17 | 12 |
| Zhao ^70^ | 2018 | MDR | Mfx+Z+Eth+H(high dose)+ETM+TRD | 18–24 months | 162 | 111 | 7 | 11 | 17 |
| Yu ^71^ | 2018 | MDR/XDR | LZD+Rfb+Km+Cm+Am+Lfx+Mfx+Pto+Cs+Trd+Pas+Cfz+Mpm+Amx/Clv | 18–24 months | 692 | 570 | 18 | 84 | 20 |
| Lotz (2) ^35^ | 2023 | RR/MDR | WHO based standard regimen | >18 months | 177 | 103 | 1 | 37 | 36 |
| Nkurunziza (2) ^52^ | 2018 | Pre-XDR | Cm + Mfx + Ofx + Lfx + Z + E + Pas + Cfz + Eto + Trd + Azm + High dose H + Amx/Clv + Clr | 19 months | 26 | 6 | 2 | 6 | 11 |
| Nguyen (1) ^72^ | 2022 | Pre-XDR | BDQ + LZD + Mfx + Cfz + Cs + Z + Pto + Pas + E | 20 months | 42 | 29 | 1 | 4 | 8 |
| Nguyen (2) ^72^ | 2022 | Pre-XDR | BDQ + LZD + Mfx + Cfz + Cs + Z + Pto + Pas + E + High dose H + Km + Cm | 20 months | 57 | 46 | 1 | 3 | 7 |
| Kashongwe ^73^ | 2020 | Pre-XDR/XDR | BDQ + LZD + Am + Km + Lfx + Cfz + Pas + Cs + high dose H + Z + Pto | 20 months | 32 | 17 | 0 | 15 | 0 |
| Butov (1) ^74^ | 2020 | MDR | WHO based standard regimen | 20 months | 114 | 58 | 21 | 11 | 24 |
| Butov (2) ^74^ | 2020 | MDR | WHO based standard regimen | 20 months | 126 | 91 | 9 | 6 | 20 |
| Yunusbaeva (1) ^75^ | 2019 | MDR | WHO based standard regimen | 20 months | 94 | 44 | 28 | 7 | 4 |
| Yunusbaeva (2) ^75^ | 2019 | MDR | WHO based standard regimen | 20 months | 160 | 26 | 84 | 34 | 15 |
| Mok ^76^ | 2019 | MDR/Pre-XDR/XDR | DLM + LZD + Mfx + Cfz + Am + Mpm | 20 months | 49 | 40 | 3 | 3 | 1 |
| Kuhlin ^77^ | 2018 | MDR/Pre-XDR/XDR | WHO based standard regimen | 20 months | 612 | 362 | 73 | 47 | 130 |
| Lee (1) ^78^ | 2011 | MDR | Lfx+ AGs+ Pa+Cs+Z+Rfm+E+Pas+ AMC+Clr+ SMX/TMP | 20 months | 123 | 97 | 6 | 4 | 10 |
| Ghimire ^79^ | 2020 | MDR | Lfx + Km + Cs + Eto + Z | 20-24 months | 98 | 85 | 1 | 5 | 3 |
| Htun ^80^ | 2018 | MDR | Am+Z+ Lfx+Eto+Cs /Lfx+Eto+Cs+Z | 20-24 months | 330 | 269 | 3 | 48 | 9 |
| Hewison^81^ | 2018 | MDR/ Pre-XDR/XDR | BDQ + WHO based standard regimen | 20-24 months | 82 | 48 | 6 | 10 | 18 |
| Hwang (1) ^82^ | 2021 | RR/ MDR/ Pre-XDR/XDR | BDQ + WHO based standard regimen | 21 months | 119 | 90 | 2 | 18 | 9 |
| Hwang (2) ^82^ | 2021 | RR/ MDR/ Pre-XDR/XDR | DLM + WHO based standard regimen | 21 months | 141 | 116 | 4 | 13 | 7 |
| Lee (1) ^83^ | 2017 | MDR/XDR | LZD + E + Z + S + Cm + FQs + Pto + Cs + Pas | 21 months | 24 | 15 | 3 | 0 | 2 |
| Guglielmetti ^84^ | 2017 | MDR | LZD+E+Z+Am+ Cm+ Mfx+Lfx+E+Pas+Cs+ Clf+Ipm/Cln+Mpm/Clv | 21 months | 45 | 36 | 1 | 3 | 5 |
| Olaru ^85^ | 2016 | MDR | BDQ+LZD+Amc + Cfz +Cla+Cs+ Dds +E+Fq +Fus+ Imp +Inj + Pas+Pto+Z + Rfb +Sxt | 21 months | 76 | 65 | 0 | 3 | 8 |
| Cavanaugh ^86^ | 2015 | MDR | Z+FQs+Cs+Pto | 21 months | 200 | 118 | 30 | 18 | 26 |
| Jo ^87^ | 2013 | MDR | WHO based standard regimen | 21 months | 48 | 20 | 20 | 1 | 3 |
| Lee (2) ^78^ | 2011 | MDR | Mfx+ AGs+ Pto+Cs+Z+Rfm+E+Pas+ AMC+Clr+ SMX/TMP | 22 months | 48 | 40 | 5 | 1 | 2 |
| Choi ^88^ | 2023 | MDR | WHO based standard regimen | 23 months | 1082 | 754 | 42 | 148 | 137 |
| Dong^89^ | 2024 | MDR | BDQ + LZD + Cfz + Lfx/Mfx + Pa + Pas + Cs + Z | 24 months | 143 | 96 | 47 | 0 | 0 |
| Lecai ^90^ | 2023 | RR/ MDR/ Pre-XDR/XDR | LZD + Lfx/Mfx + Pas + Z + Am + Cm + Pto + Amx/Clv + Clr | 24 months | 261 | 186 | NM | 1 | 21 |
| Zheng ^91^ | 2022 | MDR/Pre-XDR | Bdq + Lzd + Cfz + Lfx/Mfx + Am + Pto + Cs + Z + E | 24 months | 197 | 156 | 38 | 1 | 2 |
| Vambe ^92^ | 2020 | MDR/ Pre-XDR/XDR | BDQ + DLM + WHO based standard regimen | 24 months | 352 | 140 | 3 | 51 | 2 |
| Padayatchi (1) ^93^ | 2020 | Pre-XDR/XDR | BDQ + LZD + Mfx/Lfx + Pas + Z + Cfz + Cs/Trd | 24 months | 151 | 96 | 7 | 26 | 22 |
| Padayatchi (2) ^93^ | 2020 | Pre-XDR/XDR | BDQ + LZD + Cm/Km/Am + Mfx/Lfx + Z+ E + Pas + Eto + Cs/Trd + Cfz | 24 months | 105 | 33 | 12 | 43 | 7 |
| Olayanju (1) ^94^ | 2020 | MDR/ Pre-XDR/XDR | BDQ + LZD + Km + Trd + Z + Pas + Mfx + Lfx + High dose H + Eto + E + Cfz + Cm | 24 months | 82 | 52 | NM | NM | NM |
| Olayanju (2) ^94^ | 2020 | MDR/ Pre-XDR/XDR | BDQ + DLM + LZD + Km + Tr d + Z + Pas + Mpm + Mfx + Lfx + Lzd + High dose H + Eto + E + Cfz + Cm | 24 months | 40 | 27 | NM | NM | NM |
| Li ^95^ | 2020 | MDR/XDR | Am + FQs + Cs + Pto + Eto + Pas + T | 24 months | 398 | 101 | 64 | 10 | 23 |
| Gao ^96^ | 2020 | MDR/ Pre-XDR/XDR | BDQ + LZD + Cfz + Mfx + Lfx + Pto + Pas + Am + Cm + Z | 24 months | 177 | 151 | NM | 3 | NM |
| Xu ^97^ | 2017 | MDR | WHO based standard regimen | 24 months | 1542 | 734 | 279 | 133 | 176 |
| Lee (2) ^83^ | 2017 | MDR/XDR | BDQ + DLM + LZD + E + Z + S + Cm + FQs + Pto + Cs + Pas + High dose H + Rfb | 24 months | 52 | 42 | 3 | 2 | 2 |
| Alene (1) ^98^ | 2017 | MDR | Km/Am/Cm + Lfx/Ofx/Mfx + Pas + Pto + Z +Clr +E + Cs | 24 months | 471 | 272 | 60 | 13 | 126 |
| Jain ^99^ | 2014 | MDR | Km+Ofx/Lvx+Eto+Z+E+Cs/ Ofx+ Eto+ E +Cs | 24 months | 130 | 58 | 17 | 25 | 30 |
| Tang ^100^ | 2013 | XDR | WHO based standard regimen | 24 months | 169 | 22 | 124 | 8 | 10 |
| Alene (2) ^98^ | 2017 | XDR | Km/Am/Cm + Lfx/Ofx/Mfx + Pas + Pto + Z +Clr +E + Cs | 30 months | 10 | 3 | 0 | 3 | 4 |
| Kumar ^101^ | 2023 | MDR | Standardized regimen | 48 months | 95 | 49 | 3 | 20 | 22 |
| **Overall Treatment outcomes with ≥ 18 months regimens** | | | | | **17308** | **8673 (50.1%)** | **1229 (7.1%)** | **1281 (7 .4%)** | **1773 (10 .2%)** |
| **MIX DURATION** | | | | | | | | | |
| Prajapati ^102^ | 2017 | XDR | LZD + Cm+Pas+Mfx+high dose H+ Cfz+Amx/Clv / LZD + Pas+Mfx+high dose H+Cfz+Amx/Clv | (6-12)+18 months | 112 | 29 (25 .9%) | 10 (8 .9%) | 58 (51 .8%) | 11 (9 .8%) |
| **REGIMENS DURATION NOT AVAILABLE** | | | | | | | | | |
| Zhang ^103^ | 2024 | INHR/RR | R + H + E + Z Pas + Am/Cm + Pto + Lfx/Mfx | NM | 147 | 49 | 17 | 1 | 8 |
| Govender ^104^ | 2023 | RR/MDR | BDQ + Cfz + Lfx/Mfx + high-dose H + E + Z Lfx/Mfx + Cfz + E + Z | NM | 57 | 45 | 1 | 3 | 8 |
| Souleymane ^105^ | 2022 | MDR | LZD + Cfz + Mfx + Am + Pto + Cs + Z + E + high dose H | NM | 367 | 302 | 15 | 39 | 11 |
| Li ^106^ | 2022 | INHR | WHO based standard regimen | NM | 120 | 108 | NM | NM | NM |
| Chung ^107^ | 2022 | MDR | BDQ + DLM + WHO based standard regimen | NM | 741 | 594 | NM | NM | NM |
| Zheng (1) ^108^ | 2020 | MDR/ Pre-XDR/XDR | TB treatment for DS-TB | NM | 39 | 28 | 1 | 5 | 5 |
| Zheng (2) ^108^ | 2020 | MDR/ Pre-XDR/XDR | TB treatment for MDR TB | NM | 19 | 7 | 1 | 0 | 9 |
| Yesypenko ^109^ | 2020 | MDR/ Pre-XDR/XDR | WHO based standard regimen | NM | 373 | 138 | 20 | 70 | 49 |
| van der Walt (1) ^110^ | 2020 | MDR | WHO based standard regimen | NM | 435 | 224 | 0 | 0 | 126 |
| van der Walt (2) ^110^ | 2020 | MDR | Cs + WHO based standard regimen | NM | 278 | 167 | 0 | 0 | 41 |
| van der Walt (3) ^110^ | 2020 | MDR | Trd + WHO based standard regimen | NM | 145 | 86 | 0 | 0 | 17 |
| Lee ^111^ | 2020 | MDR/ Pre-XDR/XDR | LZD + Km + Am + Lfx + Mfx + Pto + Cs + Pas + Cfz + Cpm/Clv | NM | 74 | 68 | 4 | 1 | 1 |
| Sarin ^112^ | 2019 | MDR/ Pre-XDR/XDR | BDQ + DLM + LZD + Imp + Mfx + Cfz (6months) | NM | 53 | 25 | NM | 10 | NM |
| Waghmare ^113^ | 2017 | MDR/ Pre-XDR/XDR | Km/Cm + Eto + Cs + Lfx/Pas/Mfx + E + Z | NM | 194 | 94 | 22 | 39 | 23 |
| Villegas ^114^ | 2016 | INHR | WHO based standard regimen | NM | 85 | 63 | 2 | 4 | 13 |

Abbreviations: DR-TB: drug resistance tuberculosis, RR: rifampin-resistant, MDR:multi drug resistance, XDR: extensively drug resistance, NM:not mentioned, Km: kanamycin, Ofx:ofloxacin, Cs:cycloserin, Eto:ethionamide, BDQ: bedaquiline, Lfx:levofloxacin, Cfz: clofazimine, Z:pyrazinamide, E:ethambutol, Am:amikacin, Cm:capromycin, Mfx: moxifloxacin, PAS:para aminosalicylic acid, LZD:linezolid, Ipm/Clm: Imipenem-cilastatin, Mpm/Clv: meropenem-clavulanic acid, H:isoniazid, Trd: terizidone, FQs: fluoroquinolones, DLM:delamanide Amx/Clv:Amoxicillin-clavulanic acid, STR : short treatment regimen, AGs: aminoglycosides, Rfm: rifampicin, SMX/TMP: Sulfamethoxazole-trimethoprim, FUS:fusidic acid, INJ: injectable drugs, Rfb:rifabutin, SXT: co-trimoxazole, Rif:rifamycin, S:streptomycin, Clr/CLa:Clarithromycin, Cpm:Chlorphenamine, Gfx: Gatifloxacin**,** Pa: Pretomanid**,** Pto: Prothionamid

**Supplementary Data 3. Quality assessment of the observational studies included in the meta-analysis (The NOS tool).**

| **Author** | **Selection** | | | | **Comparability** | | **Outcome** | | | |
| --- | --- | --- | --- | --- | --- | --- | --- | --- | --- | --- |
|  | **Representativeness**  **of Exposed cohort** | **Selection of non-exposed cohort** | **Ascertainment**  **of exposure** | **Demonstration that outcome**  **of interest was not present at start of study** | **Adjust for the most important risk factors** | **Adjust for other risk factors** | **Assessment**  **of outcome** | **Follow-up length** | **Loss**  **to follow-up**  **rate** | **Total**  **quality**  **score** |
| Dong 2024 | 1 | 0 | 1 | 0 | 1 | 1 | 1 | 1 | 1 | 7 |
| Mikiashvili 2024 | 1 | 0 | 1 | 0 | 1 | 1 | 1 | 1 | 1 | 7 |
| Fan 2023 | 1 | 0 | 1 | 0 | 1 | 1 | 1 | 1 | 1 | 7 |
| Lecai 2023 | 1 | 0 | 1 | 0 | 1 | 1 | 1 | 1 | 1 | 7 |
| Prince 2023 | 1 | 0 | 1 | 0 | 1 | 1 | 1 | 1 | 1 | 7 |
| Choi 2023 | 1 | 0 | 1 | 0 | 1 | 1 | 1 | 1 | 1 | 7 |
| Govender 2023 | 1 | 0 | 1 | 0 | 1 | 1 | 1 | 1 | 1 | 7 |
| Suvendu Kumar 2023 | 1 | 0 | 1 | 0 | 1 | 1 | 1 | 1 | 1 | 7 |
| Lotz 2023 | 1 | 0 | 1 | 0 | 1 | 1 | 1 | 1 | 1 | 7 |
| Massud 2022 | 1 | 0 | 1 | 0 | 1 | 1 | 1 | 1 | 1 | 7 |
| Chung 2022 | 1 | 0 | 1 | 0 | 1 | 1 | 1 | 1 | 1 | 7 |
| Ndjeka 2022 | 1 | 0 | 1 | 0 | 1 | 1 | 1 | 1 | 1 | 7 |
| Zheng 2022 | 1 | 0 | 1 | 0 | 1 | 1 | 1 | 1 | 1 | 7 |
| Huerga 2022 | 1 | 0 | 1 | 0 | 1 | 1 | 1 | 1 | 1 | 7 |
| Zhang 2022 | 1 | 0 | 1 | 0 | 1 | 1 | 1 | 1 | 1 | 7 |
| Velayutham 2022 | 1 | 0 | 1 | 0 | 1 | 1 | 1 | 1 | 1 | 7 |
| Hwang 2021 | 1 | 0 | 1 | 0 | 1 | 1 | 1 | 1 | 1 | 7 |
| Sidamo 2021 | 1 | 0 | 1 | 0 | 1 | 1 | 1 | 1 | 1 | 7 |
| Sun 2021 | 1 | 0 | 1 | 0 | 1 | 1 | 1 | 1 | 1 | 7 |
| Koirala 2021 | 1 | 0 | 1 | 0 | 1 | 1 | 1 | 1 | 1 | 7 |
| Auchynka 2021 | 1 | 0 | 1 | 0 | 1 | 1 | 1 | 1 | 1 | 7 |
| Oelofse 2021 | 1 | 0 | 1 | 0 | 1 | 1 | 1 | 1 | 1 | 7 |
| Zheng 2020 | 1 | 0 | 1 | 0 | 1 | 1 | 1 | 1 | 1 | 7 |
| Gao 2020 | 1 | 0 | 1 | 0 | 1 | 1 | 1 | 1 | 1 | 7 |
| Ghimire 2020 | 1 | 0 | 1 | 0 | 1 | 1 | 1 | 1 | 1 | 7 |
| Padayatchi 2020 | 1 | 0 | 1 | 0 | 1 | 1 | 1 | 1 | 1 | 7 |
| Schwœbel 2020 | 1 | 0 | 1 | 0 | 1 | 1 | 1 | 1 | 1 | 7 |
| Olayanju 2020 | 1 | 0 | 1 | 0 | 1 | 1 | 1 | 1 | 1 | 7 |
| Kang 2020 | 1 | 0 | 1 | 0 | 1 | 1 | 1 | 1 | 1 | 7 |
| Lee 2020 | 1 | 0 | 1 | 0 | 1 | 1 | 1 | 1 | 1 | 7 |
| Anh 2020 | 1 | 0 | 1 | 0 | 1 | 1 | 1 | 1 | 1 | 7 |
| van der Walt 2020 | 1 | 0 | 1 | 0 | 1 | 1 | 1 | 1 | 1 | 7 |
| Vambe 2020 | 1 | 0 | 1 | 0 | 1 | 1 | 1 | 1 | 1 | 7 |
| Yunusbaeva 2019 | 1 | 0 | 1 | 0 | 1 | 1 | 1 | 1 | 1 | 7 |
| Hewison 2018 | 1 | 0 | 1 | 0 | 1 | 1 | 1 | 1 | 1 | 7 |
| Kuhlin 2018 | 1 | 0 | 1 | 0 | 1 | 1 | 1 | 1 | 1 | 7 |
| Ndjeka 2018 | 1 | 0 | 1 | 0 | 1 | 1 | 1 | 1 | 1 | 7 |
| Htun 2018 | 1 | 0 | 1 | 0 | 1 | 1 | 1 | 1 | 1 | 7 |
| Zhao 2018 | 1 | 0 | 1 | 0 | 1 | 1 | 1 | 1 | 1 | 7 |
| Yu 2018 | 1 | 0 | 1 | 0 | 1 | 1 | 1 | 1 | 1 | 7 |
| Lee 2017 | 1 | 0 | 1 | 0 | 1 | 1 | 1 | 1 | 1 | 7 |
| Xu 2017 | 1 | 0 | 1 | 0 | 1 | 1 | 1 | 1 | 1 | 7 |
| Romanowski 2017 | 1 | 0 | 1 | 0 | 1 | 1 | 1 | 1 | 1 | 7 |
| Schechter 2017 | 1 | 0 | 1 | 0 | 1 | 1 | 1 | 1 | 1 | 7 |
| Lee 2017 | 1 | 0 | 1 | 0 | 1 | 1 | 1 | 1 | 1 | 7 |
| Alene 2017 | 1 | 0 | 1 | 0 | 1 | 1 | 1 | 1 | 1 | 7 |
| Guglielmetti 2017 | 1 | 0 | 1 | 0 | 1 | 1 | 1 | 1 | 1 | 7 |
| Villegas 2016 | 1 | 0 | 1 | 0 | 1 | 1 | 1 | 1 | 1 | 7 |
| Lee 2016 | 1 | 0 | 1 | 0 | 1 | 1 | 1 | 1 | 1 | 7 |
| Jeong 2015 | 1 | 0 | 1 | 0 | 1 | 1 | 1 | 1 | 1 | 7 |
| Bonnet 2015 | 1 | 0 | 1 | 0 | 1 | 1 | 1 | 1 | 1 | 7 |
| Cavanaugh 2015 | 1 | 0 | 1 | 0 | 1 | 1 | 1 | 1 | 1 | 7 |
| Velásquez 2014 | 1 | 0 | 1 | 0 | 1 | 1 | 1 | 1 | 1 | 7 |
| Chien 2014 | 1 | 0 | 1 | 0 | 1 | 1 | 1 | 1 | 1 | 7 |
| Gegia 2012 | 1 | 0 | 1 | 0 | 1 | 1 | 1 | 1 | 1 | 7 |
| Rodriguez 2012 | 1 | 0 | 1 | 0 | 1 | 1 | 1 | 1 | 1 | 7 |
| Lee 2011 | 1 | 0 | 1 | 0 | 1 | 1 | 1 | 1 | 1 | 7 |
| Yesypenko 2020 | 0 | 0 | 1 | 0 | 1 | 1 | 1 | 1 | 1 | 6 |
| Tang 2013 | 0 | 0 | 1 | 0 | 1 | 1 | 1 | 1 | 1 | 6 |
| Zhang 2024 | 1 | 0 | 1 | 0 | 0 | 0 | 1 | 1 | 1 | 5 |
| Aslan 2023 | 1 | 0 | 1 | 0 | 0 | 0 | 1 | 1 | 1 | 5 |
| Kwon 2023 | 1 | 0 | 1 | 0 | 0 | 0 | 1 | 1 | 1 | 5 |
| Burham 2022 | 1 | 0 | 1 | 0 | 0 | 0 | 1 | 1 | 1 | 5 |
| Padmapriyadarsini 2022 | 1 | 0 | 1 | 0 | 0 | 0 | 1 | 1 | 1 | 5 |
| Nguyen 2022 | 1 | 0 | 1 | 0 | 0 | 0 | 1 | 1 | 1 | 5 |
| Li 2022 | 1 | 0 | 1 | 0 | 0 | 0 | 1 | 1 | 1 | 5 |
| Souleymane 2022 | 1 | 0 | 1 | 0 | 0 | 0 | 1 | 1 | 1 | 5 |
| Kumari 2022 | 1 | 0 | 1 | 0 | 0 | 0 | 1 | 1 | 1 | 5 |
| Kumar G 2022 | 1 | 0 | 1 | 0 | 0 | 0 | 1 | 1 | 1 | 5 |
| Nguyen 2022 | 1 | 0 | 1 | 0 | 0 | 0 | 1 | 1 | 1 | 5 |
| Avaliani 2021 | 1 | 0 | 1 | 0 | 0 | 0 | 1 | 1 | 1 | 5 |
| Kwon 2021 | 1 | 0 | 1 | 0 | 0 | 0 | 1 | 1 | 1 | 5 |
| Indarti 2020 | 1 | 0 | 1 | 0 | 0 | 0 | 1 | 1 | 1 | 5 |
| Kashongwe 2020 | 1 | 0 | 1 | 0 | 0 | 0 | 1 | 1 | 1 | 5 |
| Li 2020 | 1 | 0 | 1 | 0 | 0 | 0 | 1 | 1 | 1 | 5 |
| Butov 2020 | 1 | 0 | 1 | 0 | 0 | 0 | 1 | 1 | 1 | 5 |
| Barvaliya 2020 | 1 | 0 | 1 | 0 | 0 | 0 | 1 | 1 | 1 | 5 |
| Riccardi 2019 | 1 | 0 | 1 | 0 | 0 | 0 | 1 | 1 | 1 | 5 |
| Mok 2019 | 1 | 0 | 1 | 0 | 0 | 0 | 1 | 1 | 1 | 5 |
| Sarin 2019 | 1 | 0 | 1 | 0 | 0 | 0 | 1 | 1 | 1 | 5 |
| Bastard 2019 | 1 | 0 | 1 | 0 | 0 | 0 | 1 | 1 | 1 | 5 |
| Nkurunziza 2018 | 1 | 0 | 1 | 0 | 0 | 0 | 1 | 1 | 1 | 5 |
| Prajapati 2017 | 1 | 0 | 1 | 0 | 0 | 0 | 1 | 1 | 1 | 5 |
| Kim 2017 | 1 | 0 | 1 | 0 | 0 | 0 | 1 | 1 | 1 | 5 |
| Waghmare 2017 | 1 | 0 | 1 | 0 | 0 | 0 | 1 | 1 | 1 | 5 |
| Olaru 2016 | 1 | 0 | 1 | 0 | 0 | 0 | 1 | 1 | 1 | 5 |
| Lew 2016 | 1 | 0 | 1 | 0 | 0 | 0 | 1 | 1 | 1 | 5 |
| Wang 2014 | 1 | 0 | 1 | 0 | 0 | 0 | 1 | 1 | 1 | 5 |
| Hoa 2014 | 1 | 0 | 1 | 0 | 0 | 0 | 1 | 1 | 1 | 5 |
| Mpagama 2013 | 1 | 0 | 1 | 0 | 0 | 0 | 1 | 1 | 1 | 5 |
| Jo 2013 | 1 | 0 | 1 | 0 | 0 | 0 | 1 | 1 | 1 | 5 |
| Jain 2013 | 1 | 0 | 1 | 0 | 0 | 0 | 1 | 1 | 1 | 5 |
| Bendayan 2010 | 1 | 0 | 1 | 0 | 0 | 0 | 1 | 1 | 1 | 5 |
| Farley 2011 | 1 | 0 | 1 | 0 | 0 | 0 | 1 | 1 | 1 | 4 |

**Supplementary Data 4. Quality assessment of the experimental studies included in the meta-analysis (the Cochrane tool)**

| **Author** | **Random sequence**  **generation** | **Allocation concealment** | **Blinding of participants and**  **personnel** | **Blinding of outcome**  **assessment** | **Incomplete outcome**  **data** | **Selective reporting** | **Other bias** |
| --- | --- | --- | --- | --- | --- | --- | --- |
| Diacon 2014 | Low risk | Low risk | Low risk | Low risk | Low risk | Low risk | Low risk |
| Yao 2023 | Low risk | High risk | High risk | High risk | Low risk | Low risk | Low risk |
| Esmail 2022 | Low risk | High risk | High risk | High risk | Low risk | Low risk | Low risk |
| Du 2019 | Low risk | High risk | High risk | High risk | Low risk | Low risk | Low risk |
| Duan 2019 | Low risk | High risk | High risk | High risk | Low risk | Low risk | Low risk |
| Nunn 2019 | Low risk | High risk | High risk | High risk | Low risk | Low risk | Low risk |
| Kang 2019 | Low risk | High risk | High risk | High risk | Low risk | Low risk | Low risk |
| Tang 2014 | Low risk | High risk | High risk | High risk | Low risk | Low risk | Low risk |
| Goodall 2022 | Low risk | High risk | High risk | High risk | Low risk | Low risk | Low risk |
| Mok 2022 | Low risk | High risk | High risk | High risk | Low risk | Low risk | Low risk |
| Nyang’wa 2022 | Low risk | High risk | High risk | High risk | Low risk | Low risk | Low risk |
| Qiujing 2020 | High risk | High risk | High risk | High risk | Low risk | Low risk | Low risk |
| Conradie2022 | Low risk | Low risk | Low risk | Low risk | Low risk | Low risk | Low risk |
| Conradie2020 | High risk | High risk | High risk | High risk | Low risk | Low risk | Low risk |
| Groote-Bidlingmaier 2019 | Low risk | Low risk | Low risk | Low risk | Low risk | Low risk | Low risk |

**Supplementary Data 5. Summary of results based on type of DR-TB (Observational studies)**

| **DR-TB** | **Regimens** | **Favorable outcome % (CI)** | **Unfavorable outcome % (CI)** | **Failure % (CI)** | **Died % (CI)** | **LTFU % (CI)** |
| --- | --- | --- | --- | --- | --- | --- |
| Mono-resistance | Overall | 82.92 (78.67, 87.17) | 15.81 (13.25, 18.37) | 5.14 (1.10, 9.17) | 6.97 (3.73, 10.21) | 8.91 (5.68, 12.14) |
|  | Contain FQ | 84.70 (79.08, 90.31) | 16.84 (11.82, 21.87) | 7.37 (-0.98, 15.72) | 8.54 (2.30, 14.79) | 10.00 (9.28, 10.72) |
|  | Without FQ | 81.14 (72.71, 89.58) | 14.92 (10.21, 19.63) | 2.95 (1.71, 4.18) | 5.69 (3.43, 7.94) | 8.61 (4.71, 12.51) |
| RR/MDR | Overall | 68.41 (64.55, 72.26) | 29.00(25.29,32.72) | 9.26(5.16,13.35) | 9.46(7.18,11.74) | 12.64(10.25,15.03) |
|  | Contain BDQ/DLM/LZD | 73.93 (69.52, 78.34) | 24.34(18.81,29,88) | 6.71(0.04,13.37) | 10.10(7.22,12.97) | 10.26(7.77,12.74) |
|  | Without BDQ/DLM/LZD | 64.73 (59.35, 70.11) | 31.88 (27.27, 36.49) | 10.86(5.46,16.26) | 9.05(5.65,12.45) | 14.03(10.95,17.10) |
| Pre-XDR/XDR | Overall | 54.40(42.32,66.48) | 43.92(32.46,55.38) | 17.94(0.19,35.68) | 20.22(11.66,28.79) | 15.92(9.58,21.98) |
|  | Contain BDQ/DLM/LZD | 53.68(38.88,68.47) | 43.92(29.63,58.21) | 12.91(-3.76,29.58) | 23.34(12.47,34.21) | 13.68(10.11,17.24) |
|  | Without BDQ/DLM/LZD | 55.54(30.06,81.02) | 43.92(19.84,68.00) | 40.54(-23.83,104.90) | 15.32(8.34,22.31) | 19.48(0.03,38.92) |
| MDR/pre-XDR/XDR | Overall | 70.60(67.42,73.78) | 27.06(23.76,30.37) | 6.97 (5.17, 8.77) | 9.76 (6.84, 12.68) | 10.62 (8.50, 12.74) |
|  | Contain BDQ/DLM/LZD | 73.41(70.40,76.42) | 24.42 (19.87, 28.97) | 6.78 (3.90, 9.66) | 7.90 (3.65, 12.14) | 9.23 (6.32, 12.14) |
|  | Without BDQ/DLM/LZD | 63.90 (58.79, 69.02) | 33.35 (28.13, 38.58) | 7.37 (5.10, 9.64) | 13.60 (9.12, 18.09) | 13.38 (10.16, 16.59) |

RR; rifampicin resistant; MDR: multidrug-resistant; XDR: extensively drug-resistant; DR-TB: drug-resistant tuberculosis, LTFU: lost to follow-up, CI: confidence interval


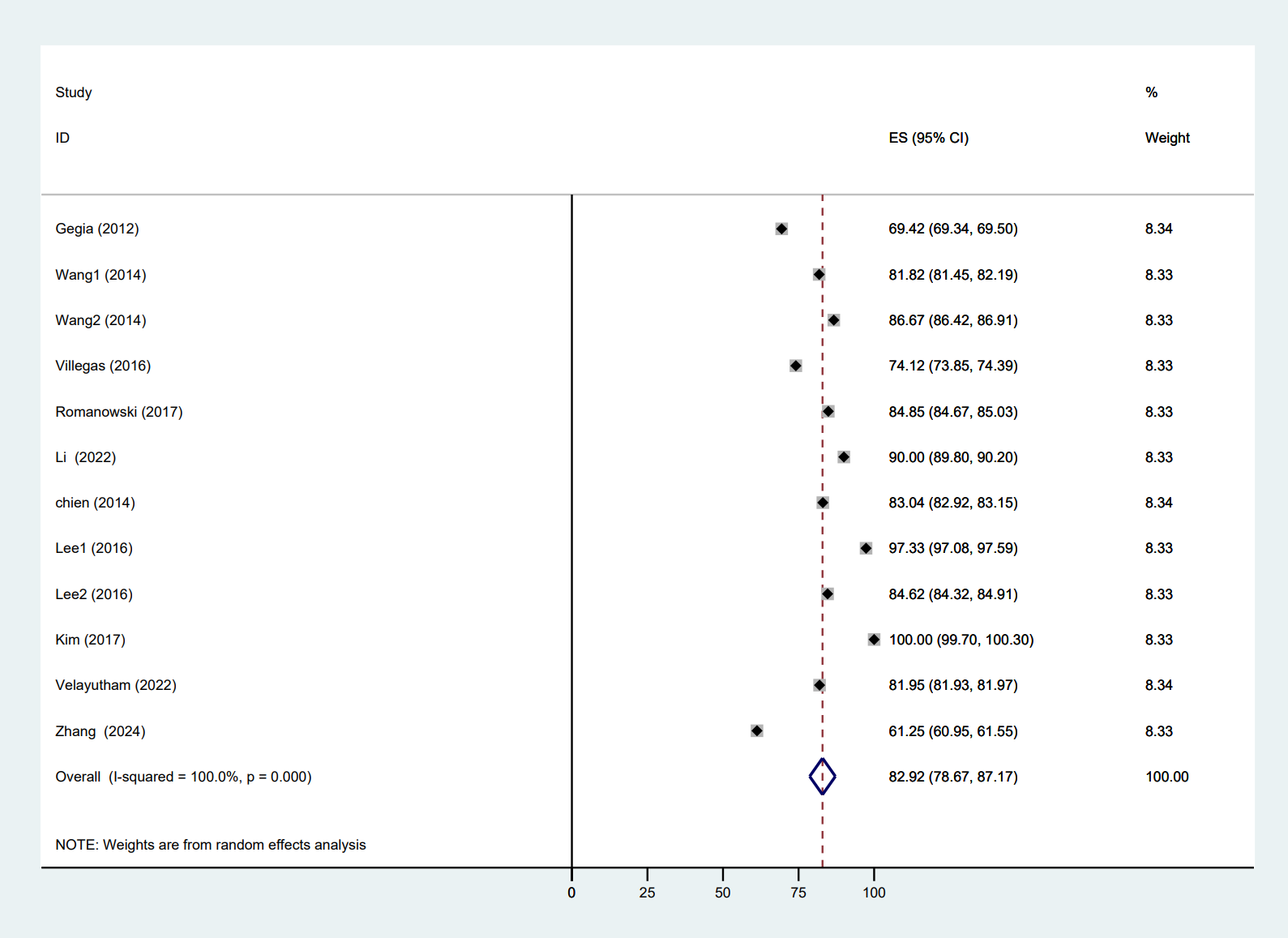


**Supplementary Data 6. Pooled favorable outcome in patients with mono-resistant Tuberculosis in observational studies**


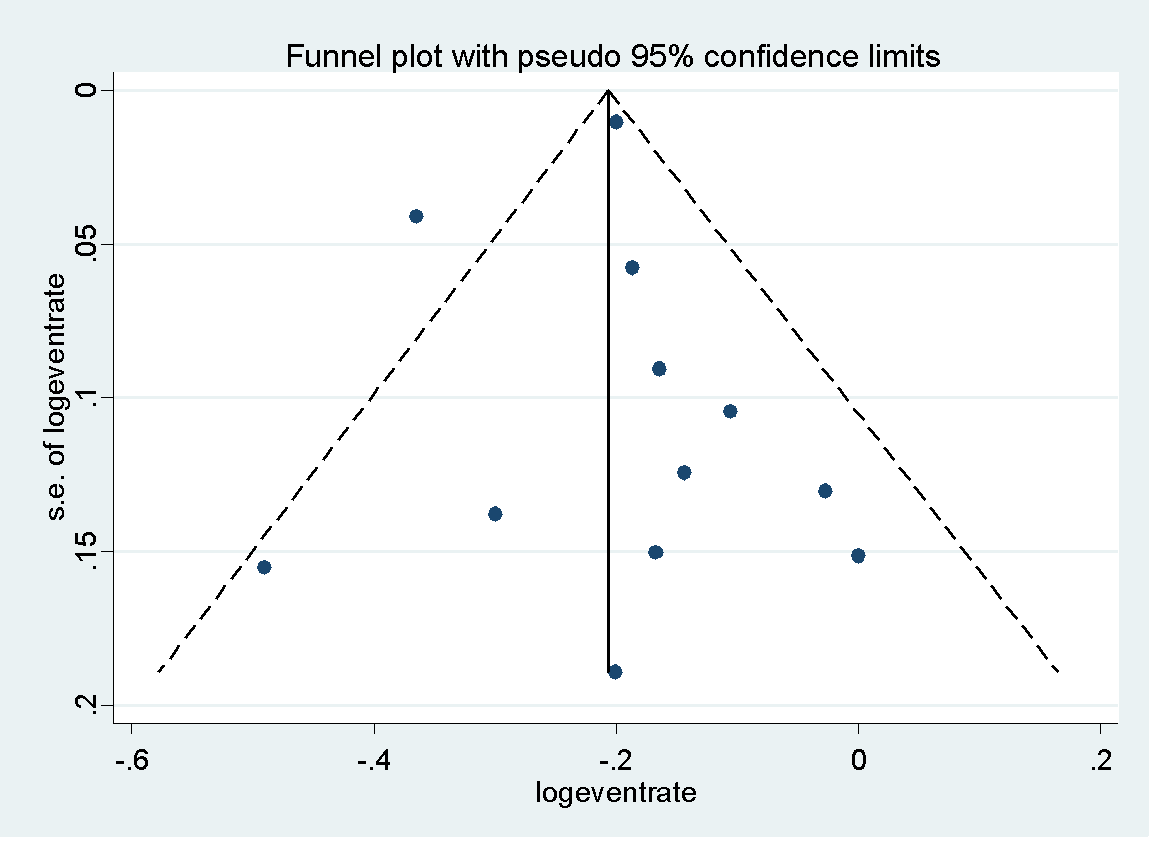


**Supplementary Data 7. Funnel plot of favorable outcome in patients with mono-resistant Tuberculosis in observational studies (p value Begg: 0.53, Egger: 0.93)**


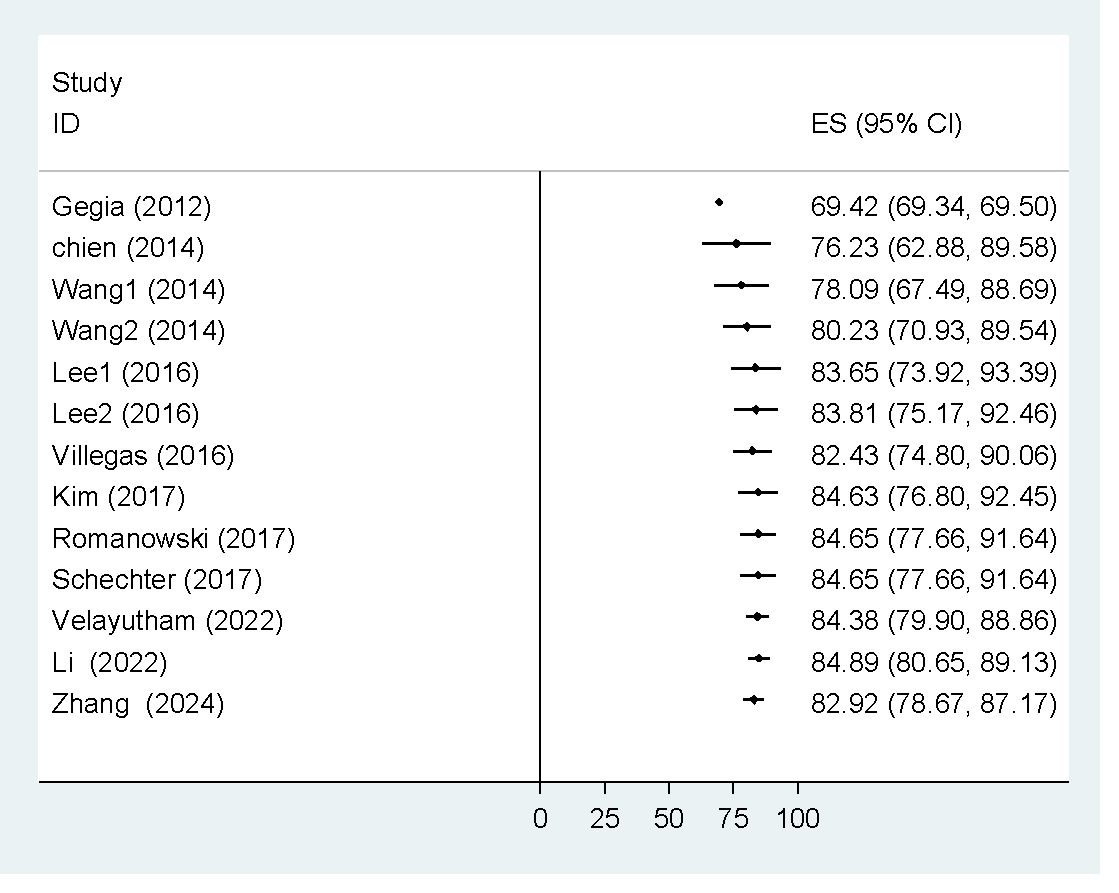


**Supplementary Data 8. Trend of favorable outcome in patients with mono-resistant Tuberculosis in observational studies**

 **Supplementary Data 9. Pooled favorable outcome in patients with Rifampicin Resistant/Multidrug-resistant-Tuberculosis in observational studies**

**Supplementary Data 10. Funnel plot of favorable outcome in patients with in observational Rifampicin Resistant/Multidrug-resistant-Tuberculosis studies (p value Begg: 0.2, Egger: 0.6)**

**Supplementary Data 11. Trend of favorable outcome in patients with Rifampicin-resistant/Multidrug-resistant Tuberculosis in observational studies**


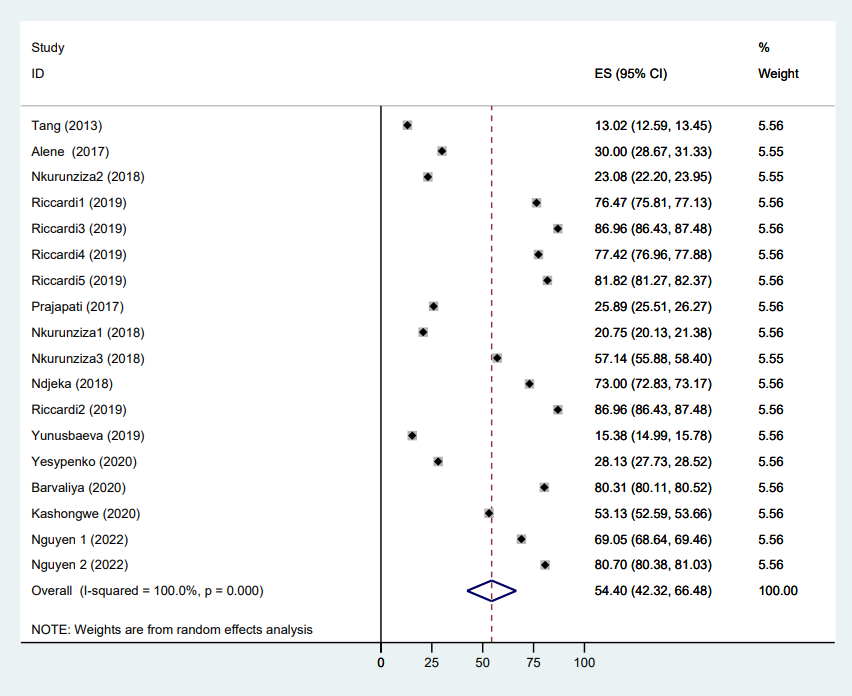


**Supplementary Data 12. Pooled favorable outcome in patients with Pre-extensively drug-resistant (XDR) and XDR Tuberculosis in observational studies**


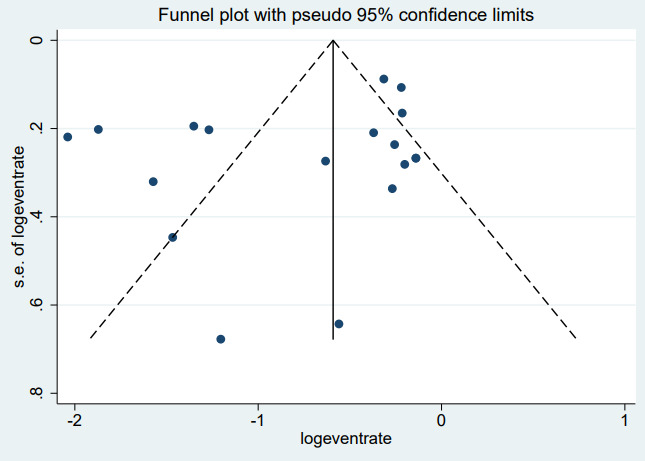


**Supplementary Data 13. Funnel plot of favorable outcome in patients with Pre-extensively drug-resistant (XDR) and XDR Tuberculosis in observational studies (p value Begg: 0.27, Egger: 0.14)**


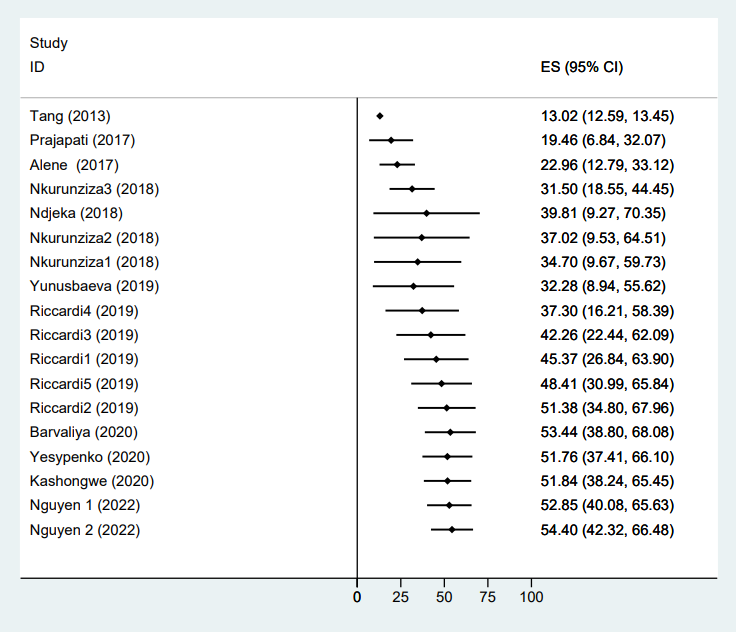


**Supplementary Data 14. Trend of favorable outcome in patients with** **Pre-extensively drug-resistant (XDR) and XDR Tuberculosis in observational studies**


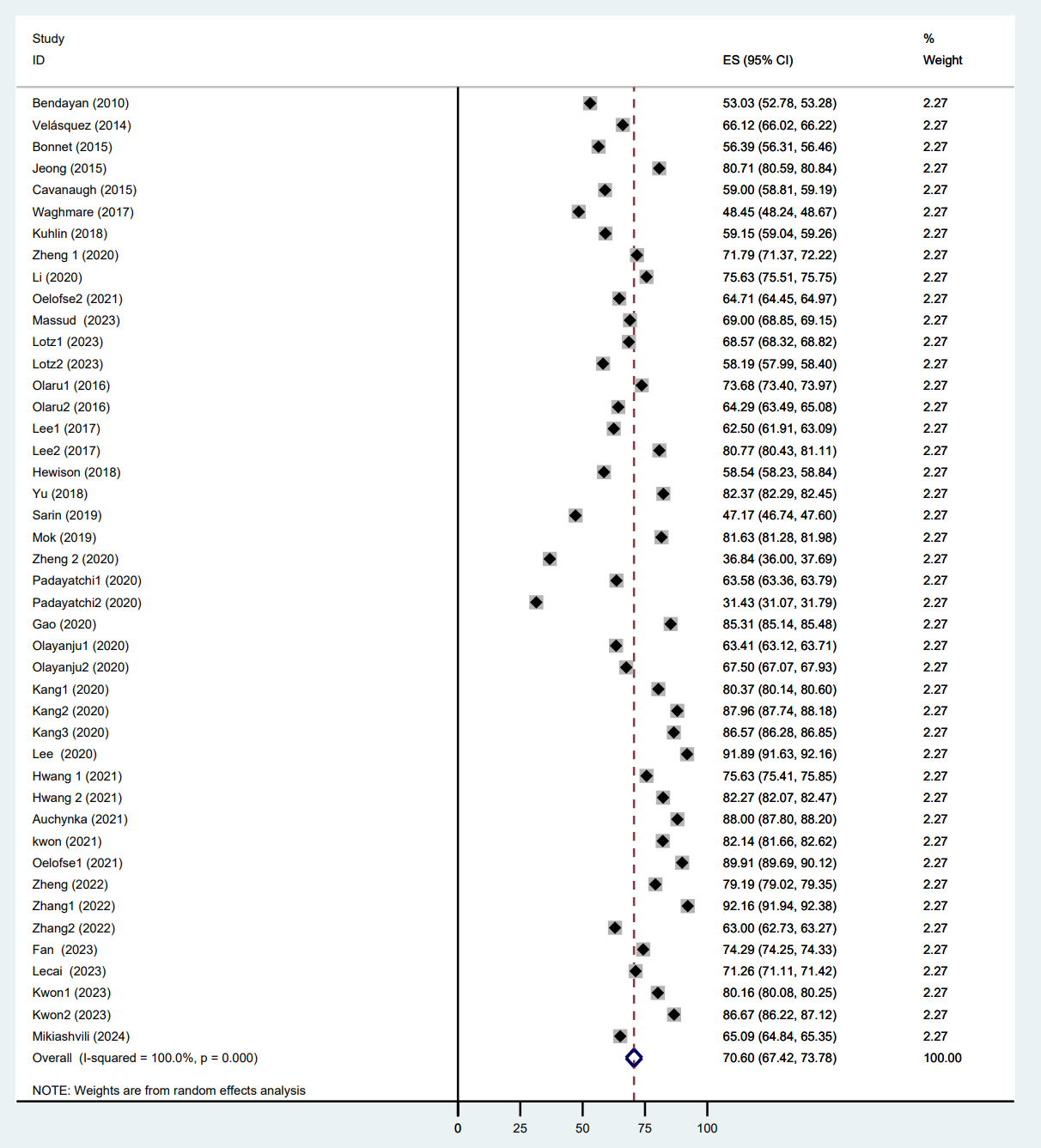


**Supplementary Data 15. Pooled favorable outcome in patients with Multidrug-resistant/ Pre-extensively drug-resistant (XDR)/XDR in observational studies**


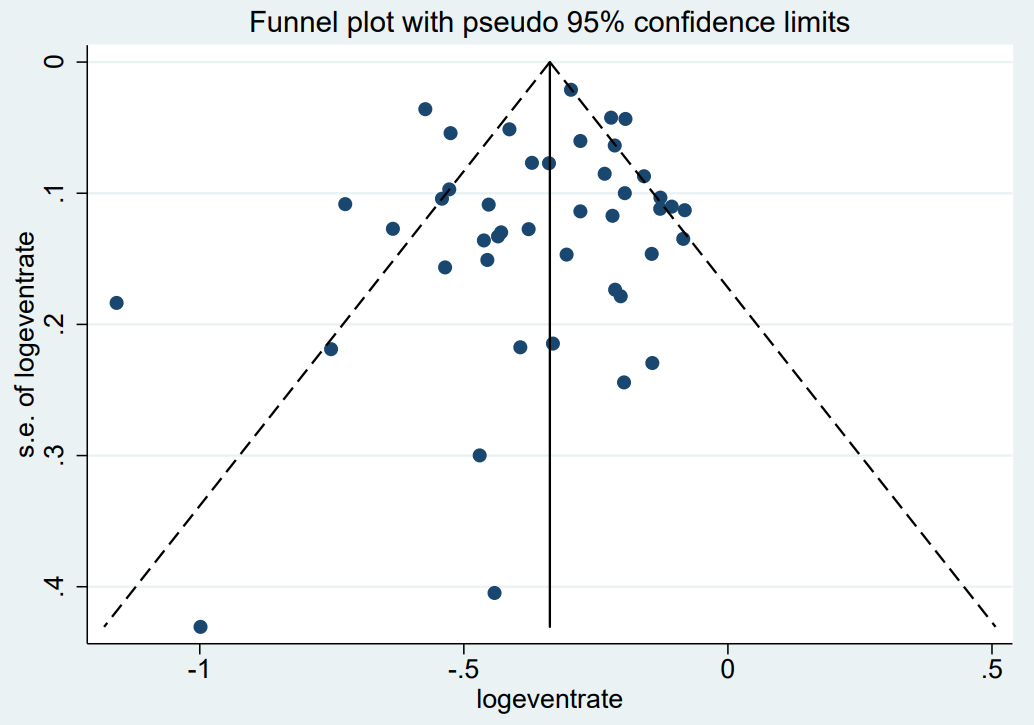


**Supplementary Data 16. Funnel plot of favorable outcome in patients with Multidrug-resistant/ (Pre-extensively drug-resistant (XDR)/XDR Tuberculosis in observational studies (p value Begg: 0.13, Egger: 0.55)**


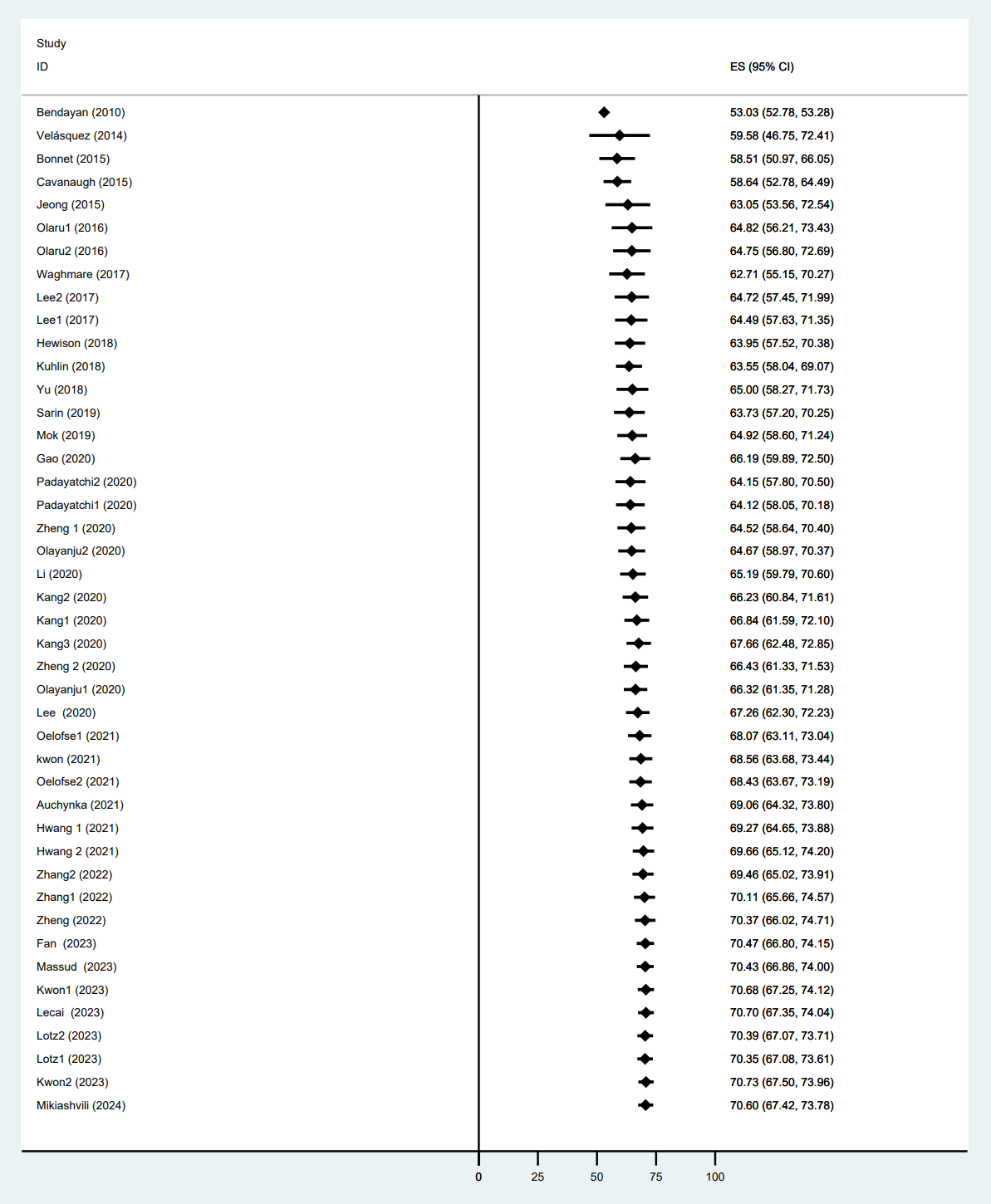


**Supplementary Data 17. Trend of favorable outcome in patients with Multidrug-resistant/Pre-extensively drug-resistant (XDR)/XDR Tuberculosis in observational studies. The Massud study (2023) used the new World Health Organization definitions 2021.**


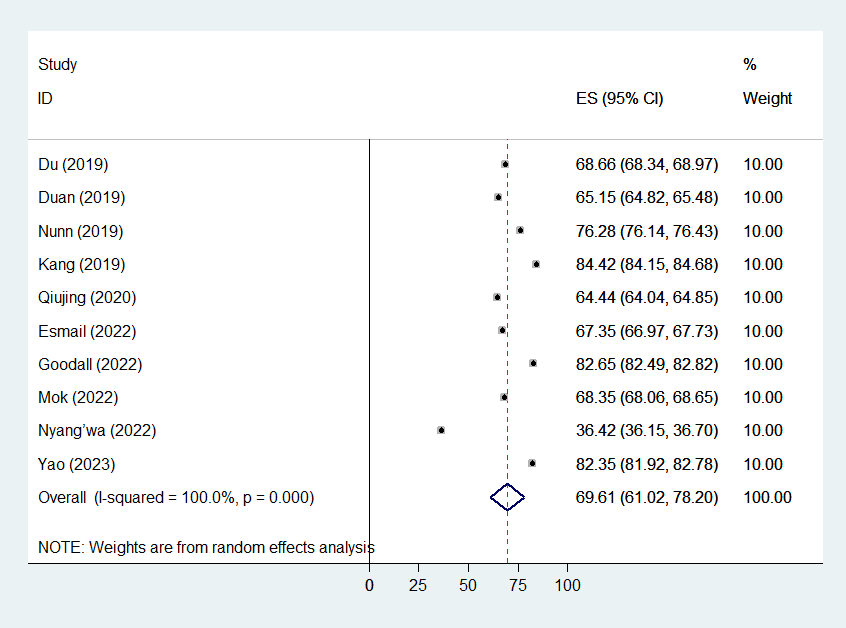


**Supplementary Data 18. Pooled favorable outcome in patients with Rifampicin Resistant/Multidrug-resistant Tuberculosis in experimental studies**


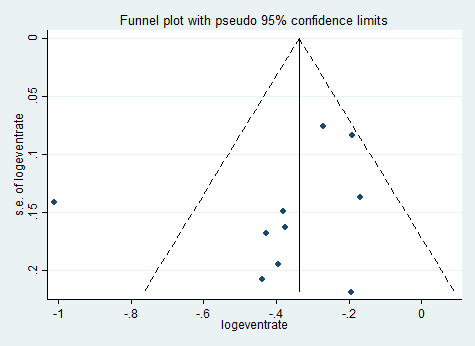


**Supplementary Data 19. Funnel plot of favorable outcome in patients with Rifampcin-resistant/Multidrg-resistant Tuberculosis in experimental studies (p value Begg: 0.30, Egger: 0.34)**


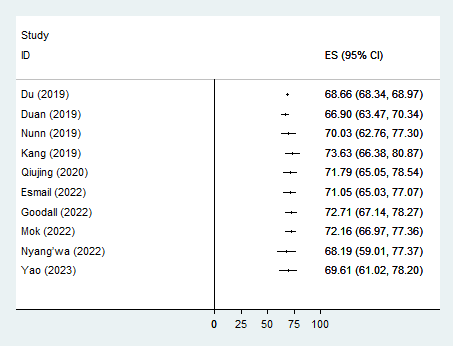


**Supplementary Data 20. Trend of favorable outcome in patients with Rifampicin-resistant/Multidrug-resistant Tuberculosis in experimental studies**
